# The Interplay Between Autophagy and Apoptosis in the Mechanisms of Action of Stilbenes in Cancer Cells

**DOI:** 10.3390/antiox14030339

**Published:** 2025-03-13

**Authors:** Kamila Siedlecka-Kroplewska, Zbigniew Kmiec, Michal Aleksander Zmijewski

**Affiliations:** 1 Department of Histology, Medical University of Gdansk, 80-211 Gdansk, Poland; michal.zmijewski@gumed.edu.pl; 2 Department of Anatomy and Histology, School of Medicine, University of Warmia and Mazury in Olsztyn, 10-082 Olsztyn, Poland; zbigniew.kmiec@uwm.edu.pl

**Keywords:** polyphenols, stilbenoids, stilbenes, resveratrol, pterostilbene, piceatannol, cancer cells, autophagy, apoptosis, cell death

## Abstract

Plant-based stilbenes are low-molecular-weight polyphenolic compounds that exhibit anti-oxidant, anti-microbial, anti-fungal, anti-inflammatory, anti-diabetic, cardioprotective, neuroprotective, and anti-cancer activities. They are phytoalexins produced in diverse plant species in response to stress, such as fungal and bacterial infections or excessive UV irradiation. Plant-derived dietary products containing stilbenes are common components of the human diet. Stilbenes appear to be promising chemopreventive and chemotherapeutic agents. Accumulating evidence indicates that stilbenes are able to trigger both apoptotic and autophagic molecular pathways in many human cancer cell lines. Of note, the molecular crosstalk between autophagy and apoptosis under cellular stress conditions determines the cell fate. The autophagy and apoptosis relationship is complex and depends on the cellular context, e.g., cell type and cellular stress level. Apoptosis is a type of regulated cell death, whereas autophagy may act as a pro-survival or pro-death mechanism depending on the context. The interplay between autophagy and apoptosis may have an important impact on chemotherapy efficiency. This review focuses on the in vitro effects of stilbenes in different human cancer cell lines concerning the interplay between autophagy and apoptosis.

## 1. Introduction

**Figure 1:**
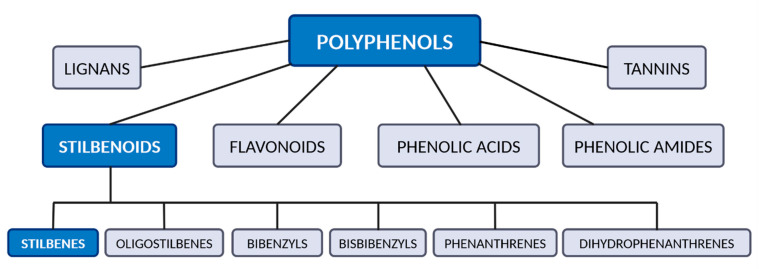
Classification of polyphenols (created in BioRender.com).

**Figure 2:**
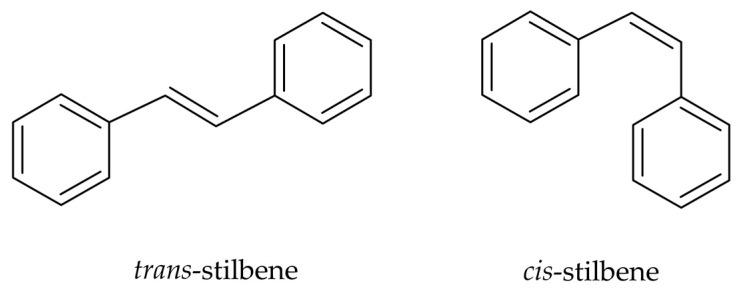
The chemical structure of stilbene.

Plant-based antioxidants, including stilbenes, have gained attention because of their beneficial biological activities and clinical potential. Stilbenes are naturally occurring low-molecular-weight compounds belonging to the group of stilbenoids (Figure 1) [1]. Stilbenoids are a subgroup of plant polyphenols. They are classified into stilbenes, oligostilbenes, bibenzyls, bisbibenzyls, phenanthrenes, and dihydrophenanthrenes (Figure 1) [1,2]. The chemical structure of stilbenoids is based on the chemical structure of stilbene (1,2-diphenylethylene; 1,2-diarylethene) comprising two aromatic rings linked by an ethylene bridge (Figure 2). The type, number, and position of substituents on the aromatic rings determine the chemical properties as well as biological activities of these compounds. Stilbenes exist in two isomeric forms: *cis* (*Z*) and *trans* (*E*). The *trans* isomers are usually more stable, more common, and exhibit higher biological activities than the *cis* isomers.

**Figure 3:**
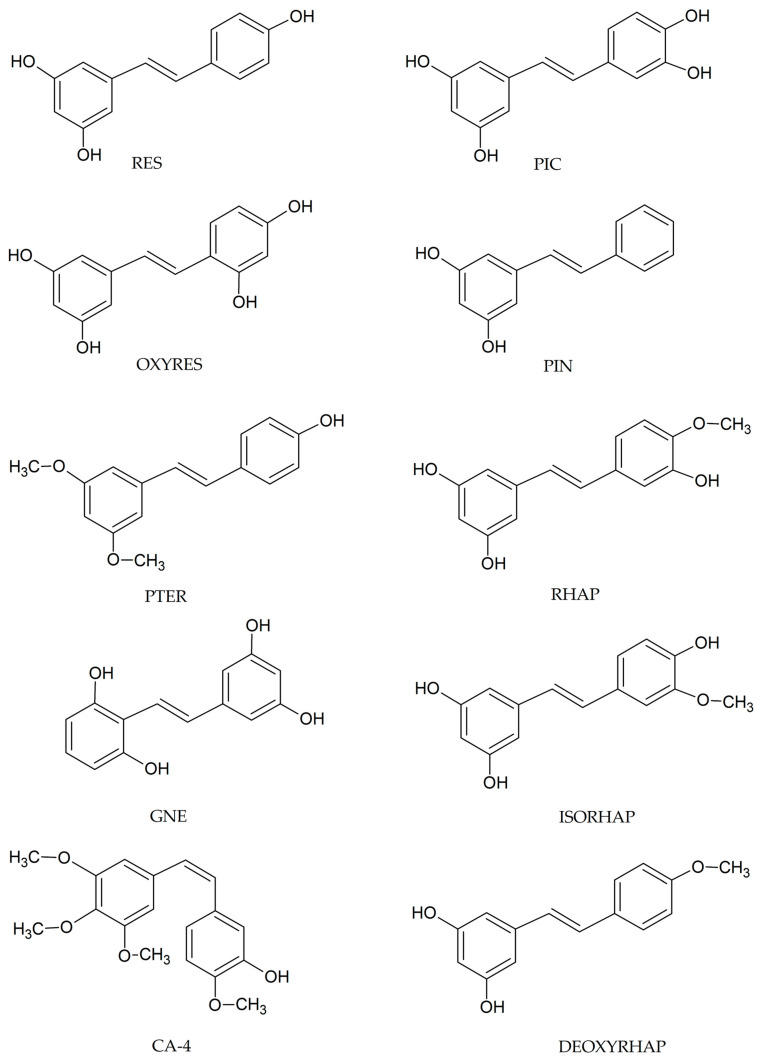
Chemical structures of selected stilbenes. RES, resveratrol; PIC, piceatannol; OXYRES, oxyresveratrol; PIN, pinosylvin; PTER, pterostilbene; RHAP, rhapontigenin; GNE, gnetol; ISORHAP, isorhapontigenin; CA-4, combrestatin A-4; DEOXYRHAP, deoxyrhapontigenin.

The examples of stilbenes include resveratrol (*trans*-3,4′,5-trihydroxystilbene; RES), pterostilbene (*trans*-3,5-dimethoxy-4′-hydroxystilbene; PTER), piceatannol (*trans*-3,3′,4′,5-tetrahydroxystilbene; PIC), oxyresveratrol (*trans*-2,3′,4,5′-tetrahydroxystilbene; OXYRES), gnetol (*trans*-2,3′,5′,6-tetrahydroxystilbene; GNE), pinosylvin (*trans*-3,5-dihydroxystilbene; PIN), rhapontigenin (*trans*-3,3′,5-trihydroxy-4′-methoxystilbene; RHAP), isorhapontigenin (*trans*-3,4′,5-trihydroxy-3′-methoxystilbene; ISORHAP), deoxyrhapontigenin (*trans*-3,5-dihydroxy-4-methoxystilbene; DEOXYRHAP), and combrestatin A-4 (*cis*-3′-hydroxy-3,4,4′-5-tetramethoxystilbene; CA-4), (Figure 3) [1,2]. Stilbenes exist as monomers and oligomers [2,3,4]. For example, ε-viniferin is a dimer of RES, whereas scirpusin B is a dimer of PIC [3,4,5]. Other examples are α-viniferin or albiraminol A, representing a trimer and a hexamer of RES, respectively [5]. Stilbenes are often isolated from plants in the form of glycosylated or prenylated derivatives [1,5]. For example, 3-*O*-β-glucoside of RES is known as piceid, whereas 3-*O*-β glucoside of PIC is called astringin [3,4,5].

Stilbenes are secondary metabolites derived from the phenylpropanoid pathway [1]. They are phytoalexins produced by plants in response to stress such as fungal and bacterial infections or excessive UV irradiation [1,2]. Stilbenes are present in diverse plant species [1]. So far, more than 1000 stilbenes have been reported [2,6]. Stilbenes are synthesized by a wide range of plant species, including Dipterocarpaceae, Cyperaceae, Gnetaceae, Pinaceae, Leguminosae, Myrtaceae, Moraceae, Fagaceae, Liliaceae, and Vitaceae [7]. They are distributed in different parts of plants, e.g., roots, rhizomes, stems, barks, leaves, seeds, and fruits [7]. They can be found in herbs and vegetables (Table 1). Plant-derived dietary products containing stilbenes are common components of the human diet (Table 1).

**Figure 4:**
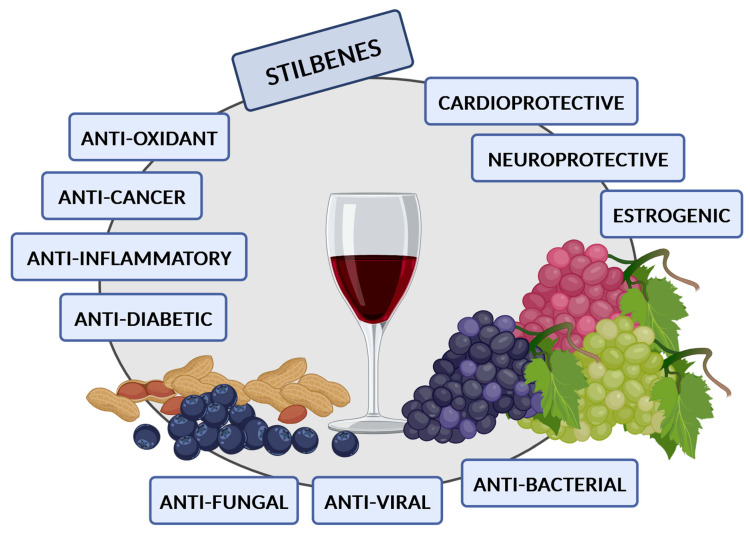
Biological activities of stilbenes (created in BioRender.com).

Stilbenes exhibit a wide range of biological properties including anti-oxidant, anti-microbial (anti-bacterial and anti-viral), anti-fungal, cardioprotective, neuroprotective, anti-diabetic, anti-inflammatory, estrogenic, and anti-cancer activities (Figure 4).

RES, PTER, and PIC are the most widely studied dietary stilbenes. Noteworthily, RES and PTER have been reported to be ingredients of ‘darakchasava’, a herbal preparation, well known in the traditional medicine of India in the treatment of heart diseases, tumors, tuberculosis, and other disorders [76]. Moreover, RES and PIC have been detected in the dried roots of *Polygonum cuspidatum*, traditionally used in Chinese and Japanese medicines as an anti-inflammatory agent [40,41,77,78,79]. PTER and OXYRES are active components of the heartwood extract of *Pterocarpus marsupium* used in traditional Ayurvedic medicine to treat diabetes mellitus [58].

Oxidative stress is implicated in many diseases [80,81]. Antioxidants may ameliorate oxidative damage of cells and help prevent or treat diseases [80,81]. By scavenging reactive oxygen species (ROS) they may also reduce oxidative stress-related adverse effects of chemotherapeutics. Numerous in vitro and in vivo studies have demonstrated that stilbenes exhibit anti-oxidant properties [82]. RES, PTER, and PIC appeared to be very effective free radical scavengers [83,84,85]. Moreover, RES has been found to increase the activity of enzymes such as superoxide dismutase and catalase involved in the anti-oxidant defense against oxidative stress [86]. RES, PTER, and PIC have been reported to inhibit peroxidation of lipids [83,87,88]. RES has been shown to modulate the gene expression of anti-oxidative enzymes superoxide dismutase 1, glutathione peroxidase 1, and pro-oxidative enzyme NADPH oxidase [89].

The main aim of this review was to provide a comprehensive analysis of the literature focusing on the in vitro effects of stilbenes in different human cancer cell lines concerning the modulation of both autophagy and apoptosis. Autophagy may act synergistically or antagonistically with apoptosis, which may have implications for cancer treatment. Therefore, the interplay between autophagy and apoptosis in cancer cells should be considered in studies focusing on the improvement of anti-cancer therapy strategies. The effect exerted by the compound is context-dependent and depends, e.g., on the type and concentration of the compound, the type of the experimental model, treatment time as well as the type of experimental methods used to study this effect. In this review, we collected the currently available published data and thoroughly analyzed mechanisms of action of stilbenes, emphasizing the aforementioned experimental conditions and providing researchers with background information for planning future research in this field.

## 2. Autophagy

Autophagy is an evolutionarily conserved catabolic process in eukaryotes [90]. In mammalian cells, it serves as a crucial pro-survival mechanism that helps cells survive under cellular stress conditions such as nutrient depletion, energy insufficiency, or hypoxia. It targets cytoplasmic constituents including damaged, misfolded, or redundant proteins, protein aggregates, and damaged, dysfunctional, or senescent cell organelles for lysosomal degradation and recycling. By recycling cellular components, autophagy helps cells maintain cellular metabolism and energy homeostasis. It protects cells from the accumulation of damaged proteins and organelles. It also plays an important role during development and differentiation as well as regulates immunity [90]. Notably, autophagy plays a key role in the removal of the nucleus during keratinocyte terminal differentiation [91]. Importantly, in post-mitotic cells, e.g., neurons, autophagy is crucial in maintaining cell functions, and defects in autophagy result in neurodegeneration and contribute to Parkinson’s, Alzheimer’s, and Huntington’s diseases [90].

**Figure 5:**
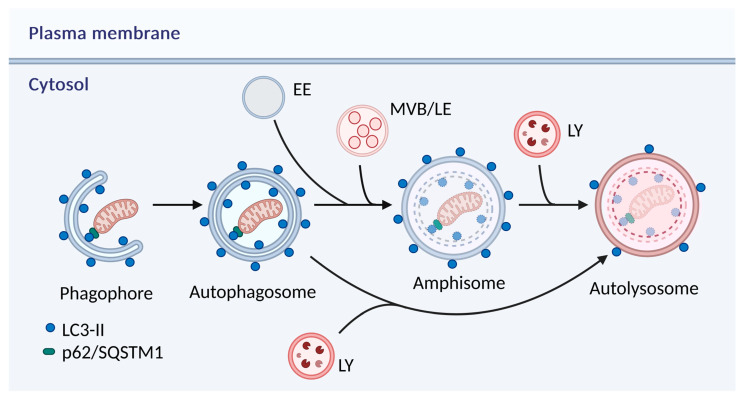
Overview of macroautophagy. EE—early endosome, MVB/LE—multivesicular body/late endosome; LY—lysosome. Created in BioRender.com.

Autophagy is classified into three types: macroautophagy, microautophagy, and chaperone-mediated autophagy (CMA) [90]. The mechanism of macroautophagy (hereafter referred to as autophagy) is based on the sequestration of the cytoplasmic material in autophagic vacuoles followed by its degradation by lysosomal enzymes [90]. After induction of autophagy, a phagophore/isolation membrane is formed which is a flat membrane cistern (Figure 5) [90,92,93]. It elongates and wraps around cytoplasmic material such as cytosol and/or organelles forming an autophagosome. Noteworthily, de novo synthesis, as well as recruitment of several cellular structures including endoplasmic reticulum (ER), Golgi complex, plasma membrane, endosomes, mitochondria, ER-mitochondria contact sites, and lipid droplets, has been suggested to contribute to the origin of the isolation membrane [93,94,95,96,97]. Recent studies indicated that an omegasome, an ER domain, is the most plausible source for the isolation membrane [94,95,96]. The formation of an autophagosome involves the following steps: nucleation, elongation, and completion. The autophagic molecular machinery recruited in the formation of the autophagosome includes many autophagy-related (ATG) proteins and is regulated by upstream signals. Autophagosomes undergo a stepwise maturation to autolysosomes, which involves fusion with early and late endosomes/multivesicular bodies, as well as lysosomes [90,92,98]. Autophagosomes can fuse with late endosomes/multivesicular bodies to form amphisomes. Autolysosomes can be formed following the fusion of autophagosomes or amphisomes with lysosomes. The outer autophagosomal membrane fuses with lysosomes [98]. Autophagosomes are double-membrane-bound vacuoles and do not contain lysosomal enzymes, whereas both amphisomes and autolysosomes are single-membrane-bound vacuoles and contain lysosomal enzymes [90,92]. Importantly, the LC3-II protein bound to both the outer and inner membrane of the autophagosome is considered an autophagy-specific marker [90]. Based on its interaction with specific cargo receptors such as sequestosome-1 (p62/SQSTM1) autophagy cargos are selectively sequestered in the autophagosome [99]. Lysosomal degradation products such as amino acids, fatty acids, monosaccharides, and nucleotides are transported to the cytosol. Lysosomes are reformed following the degradation of the sequestered material in the process of autophagic lysosome reformation (ALR) [100].

## 3. Apoptosis

Apoptosis is a type of regulated cell death that plays a crucial role in maintaining organismal homeostasis [101]. Apoptosis may be induced by many different factors including UV radiation, oxidative stress, oxygen or nutrient deficits, hormones, cytokines, growth factors deficiency, and chemotherapeutic agents [101,102,103]. In apoptosis, various signaling pathways may be involved, depending on the context, e.g., the type of inducing factor and the cell type. During apoptosis, three phases may be distinguished: induction, effector, and destruction [104]. In the induction phase, the cell receives the “death signal”, e.g., is exposed to unfavorable conditions. In the effector phase, the apoptotic signal is amplified and the execution mechanisms are triggered, whereas during the destruction phase, cellular structures are degraded.

**Figure 6:**
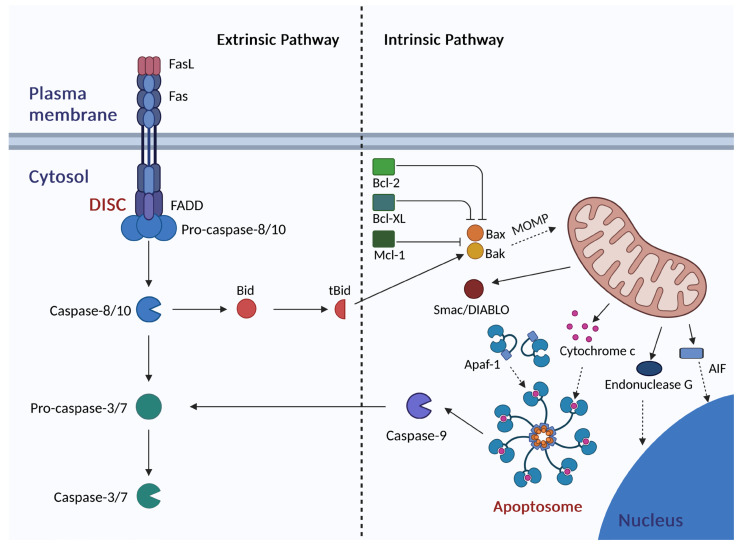
Overview of apoptosis. DISC, death-inducing signaling complex; MOMP, mitochondrial outer membrane permeabilization. Created in BioRender.com.

The best-known apoptotic molecular pathways are the extrinsic pathway and intrinsic pathway (Figure 6) [101,102,103]. The extrinsic (receptor) pathway is associated with the activation of death receptors belonging to the tumor necrosis factor (TNF) receptor superfamily such as Fas (also called CD95 or Apo-1), TNF receptor 1 (TNF-R1), TNF-related apoptosis-inducing ligand receptor 1 (TRAIL-R1; also called death receptor 4,DR-4), and TNF-related apoptosis-inducing ligand receptor 2 (TRAIL-R2; also known as death receptor 5, DR-5) (Figure 6) [101,102]. TNF receptors are transmembrane proteins and exhibit high sequence homology. The extracellular domain of the death receptors is involved in the recognition of a ligand. The cytoplasmic domain, known as the death domain (DD) interacts with adaptor proteins such as receptor-interacting protein (RIP), Fas-associated death domain (FADD), and TNF-R1 receptor-associated death domain (TRADD) [102,105]. One of the widely studied and well-known death receptors is the Fas receptor [102]. It can be found in T lymphocytes as well as in cancer cells [102,105]. FasL (CD95L) is a ligand for the Fas receptor [102]. Following binding FasL, the Fas receptor undergoes trimerization [102]. The adaptor protein FADD then binds to Fas and undergoes oligomerization. The DD domain of FADD interacts with the DD domain of Fas, whereas the death effector domain (DED) of FADD binds to the DED domain of pro-caspase-8. The resulting heterocomplex, known as the death-inducing signaling complex (DISC), enables the activation of initiator caspase-8 or initiator caspase-10 via autoproteolysis, which then triggers a cascade of proteolysis of other caspases, leading to cell death [102,103]. Activated caspase-8 cleaves the downstream effector pro-caspases such as pro-caspase-3 and pro-caspase-7, which leads to their activation [102]. Moreover, caspase-8 is able to cleave the Bid protein that acts as a link between the extrinsic and intrinsic pathways [102]. Bid is the pro-apoptotic protein belonging to the B-cell leukemia/lymphoma-2 (Bcl-2) family. The active form of Bid, called truncated Bid (tBid), translocates to the mitochondrion and induces the release of cytochrome c and other pro-apoptotic factors, thereby activating the intrinsic apoptotic pathway [102]. Importantly, the activity of caspases can be modulated by the inhibitor of apoptosis (IAP) proteins [102].

In the intrinsic (mitochondrial) apoptotic pathway, mitochondria play a key role (Figure 6) [101,102,106]. Factors leading to its induction include oxidative stress, DNA damage, growth factor deficiency, and chemotherapeutics [101,102]. Pro-apoptotic proteins Bak and Bax mediate mitochondrial outer membrane permeabilization (MOMP), which is considered the “point of no return” [101,102,106]. Bak/Bax oligomers form pores in the mitochondrial outer membrane leading to the release of cytochrome c and pro-caspases –2, -3, -9 [101,103,106,107]. The activation of Bak and Bax is inhibited by anti-apoptotic proteins belonging to the Bcl-2 family such as Bcl-2, Bcl-XL, and Mcl-1 [102,103,106]. In the cytosol, cytochrome c binds to dATP or ATP and apoptotic protease-activating factor 1 (Apaf-1) [102,107]. Apaf-1 then undergoes oligomerization. A heptameric complex is formed, to which pro-caspase-9 molecules are attached based on interactions between the caspase activation and recruitment domain (CARD) of the pro-caspase-9 and the CARD domain of Apaf-1 [108]. In the resulting multiprotein complex, called the apoptosome, the initiator pro-caspase-9 is activated via autoproteolysis, which triggers the caspase activation cascade [101,102,107]. Activated caspase-9 is able to cleave effector pro-caspases such as pro-caspase-3 and pro-caspase-7. In addition to cytochrome c and pro-caspases, other proteins involved in apoptosis are released from the mitochondrion, e.g., a second mitochondria-derived activator of caspases/direct IAP-binding protein with low pI (Smac/DIABLO), high-temperature requirement protein A2 (Omi/HtrA2), apoptosis-inducing factor (AIF), and endonuclease G [101,102,109]. Smac/DIABLO and Omi/HtrA2 are able to bind to IAP proteins, preventing IAP interactions with caspases. Smac/DIABLO interacts with XIAP, HIAP1, HIAP2, and survivin, while Omi/HtrA2 interacts with the XIAP inhibitor [103,110,111]. AIF and endonuclease G translocate to the nucleus and play a significant role in DNA fragmentation [102,109].

## 4. The Role of Autophagy and Apoptosis in Cancer Cells

Autophagy plays a dual role in carcinogenesis [90,112,113]. It acts as a double-edged sword. It may prevent or promote carcinogenesis depending on the context. Importantly, defects of the autophagy machinery resulting from deletions of genes encoding proteins involved in autophagy such as Bcl-2 interacting protein (Beclin 1), autophagy-related 5 (ATG5), autophagy-related 7 (ATG7) can initiate malignant transformation [114,115]. Accumulating evidence suggests that there is a link between mutations of autophagy genes and tumor susceptibility. Several essential autophagy genes, such as *BECN1* or *UVRAG* have been shown to function as tumor suppressors [116,117]. Thus, autophagy helps normal cells maintain homeostasis preventing tumor initiation. However, in cancer cells, autophagy also plays a pro-survival role and may support tumor progression [114,115]. Similarly to normal cells, in the case of metabolic stress related to nutrient deprivation, autophagy provides cancer cells with an energy source via the recycling of intracellular components. The elimination of damaged mitochondria lowers the risk of ROS generation and genotoxic stress. Moreover, induction of autophagy in response to chemotherapeutics helps cancer cells eliminate damaged cellular structures disturbing cellular homeostasis [113]. Autophagy has also been shown to support the survival of liver and ovarian cancer stem cells in the hypoxic and nutrient-deprived tumor microenvironment, as well as maintain their stemness [118,119]. Thus, autophagy may act as an intrinsic chemoresistance mechanism and promote the survival of cancer cells.

Noteworthily, the induction of autophagy in cancer cells may also result in cell demise. However, the role of autophagy in cell death is context-dependent. Based on the literature, the role of autophagy in cell death can be defined in the following ways: (i) autophagy-associated cell death, (ii) autophagy-mediated cell death, and (iii) autophagy-dependent cell death [120]. In the case of autophagy-associated cell death, autophagy accompanies the cell death but does not play an active executive role in this process. Autophagy-mediated cell death mediates cell death, e.g., triggers apoptosis, and inhibition of autophagy prevents cell death. Autophagy-dependent cell death is a distinct mechanism of cell death, independent of apoptosis or necrosis. The Nomenclature Committee on Cell Death has defined autophagy-dependent cell death as a distinct cell death subroutine, a form of regulated cell death that mechanistically depends on the autophagic machinery (or components thereof) [121,122]. Autophagy-dependent cell death can be retarded by pharmacological or genetic inhibition of macroautophagy [122].

Apoptosis is a type of regulated cell death that helps maintain tissue and organismal homeostasis by eliminating damaged, unnecessary cells, as well as cancer cells [123]. Cancer cells use different mechanisms to evade apoptosis. Numerous chemotherapeutic drugs used in anti-cancer therapy eliminate cancer cells by targeting apoptotic pathways [124].

Numerous studies highlight the role of inflammatory processes in carcinogenesis [125,126,127,128]. Chronic inflammation may contribute to tumor initiation and progression [125]. Targeting inflammatory processes in cancer cells has emerged as a new approach to anti-cancer therapy [125,126]. Inflammatory pathways may suppress or induce autophagy in cancer cells depending on the context [127]. On the contrary, autophagy may suppress or promote inflammation in cancer [127]. The production of cytokines related to the inflammatory process promotes the survival of cancer cells. For example, interleukin 6 produced by immune cells plays an important role in the inhibition of apoptosis in cancer cells [128]. Noteworthily, plant-derived polyphenols have been reported to influence inflammatory pathways [125]. Polyphenolic antioxidants such as RES have been found to inhibit the nuclear factor kappa B (NF-κB) activation leading to apoptosis [125]. Noteworthily, NF-κB plays an important role in many processes including inflammation, cell growth, and survival [129]. In some cancer cell types NF-κB pathway is dysregulated and constitutively active promoting cell proliferation.

## 5. The Interplay Between Autophagy and Apoptosis

Accumulating data indicates that the molecular crosstalk between autophagy and apoptosis under cellular stress conditions determines the cell fate. The autophagy and apoptosis relationship is complex and depends on the cellular context, e.g., cell type, stress stimuli/factors, and cellular stress level. Noteworthily, at low damage or a low stress level, autophagy may be sufficient to remove dysfunctional organelles or proteins, whereas at high damage or a high stress level the autophagy capacity may be exhausted and insufficient to maintain cell homeostasis. High continuous autophagic flux may be self-destructive and lead to cell death.

**Figure 7:**
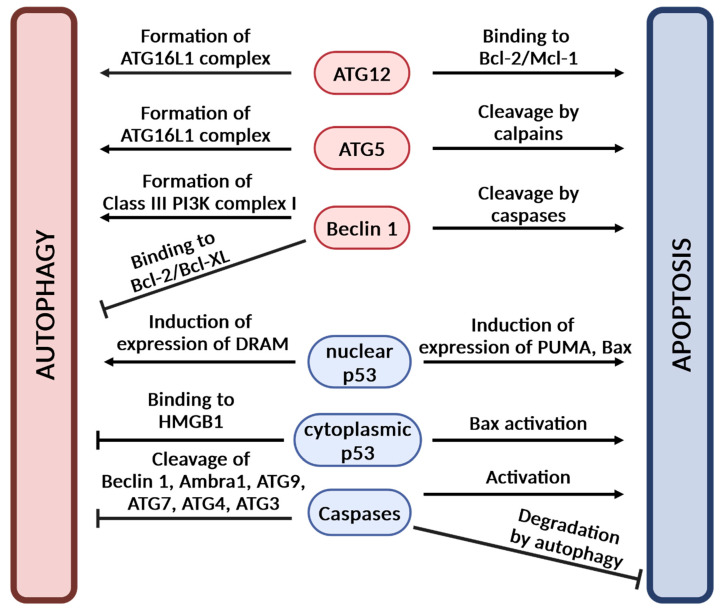
The interplay of autophagy and apoptosis. Created in BioRender.com.

Autophagy and apoptosis involve distinct molecular pathways but are interconnected by some molecules (Figure 7). Several autophagy proteins such as autophagy related 12 (ATG12) and Beclin 1 are involved in apoptosis regulation. For example, ATG12, a component of the autophagy-related 16-like 1 (ATG16L1) complex, was found to have a pro-apoptotic activity in HEK293 and HeLa cells [130]. It binds to anti-apoptotic Bcl-2 and Mcl-1 proteins leading to apoptosis [130]. Beclin 1, a component of the class III phosphatidylinositol 3-kinase (class III PI3K) complex I, can interact with anti-apoptotic Bcl-2 family members. It binds to anti-apoptotic Bcl-2 and Bcl-XL proteins, which prevents the formation of the class III PI3K complex I leading to autophagy inhibition [131,132]. Dissociation of Beclin 1 from Bcl-2/Bcl-XL leads to autophagy induction. Moreover, several proteins engaged in the autophagy pathway including Beclin 1, ATG7, activating molecule in Beclin-1-regulated autophagy 1 (Ambra1), autophagy-related 9 (ATG9), autophagy-related 4 (ATG4), and autophagy-related 3 (ATG3) were shown to be cleaved by caspases [132,133,134]. Caspase-3 was reported to cleave Beclin 1, ATG9, ATG7, ATG3, and ATG4 homologues such as ATG4A, ATG4B, ATG4C, and ATG4D [132,133]. Notably, Beclin 1 cleavage products were found to induce cytochrome c release and apoptosis [135]. In addition, ATG5 was shown to be cleaved by calpains leading to apoptosis [136]. Degradation of apoptosis-related proteins such as caspase-8 by autophagy has also been reported [132,137]. The p53 protein was demonstrated to induce growth arrest and apoptosis in response to various stress signals. However, it was also found to be involved in autophagy regulation. In response to genotoxic stress, the nuclear p53 may lead to autophagy induction via the activation of AMP-activated protein kinase (AMPK) and damage-regulated autophagy modulator (DRAM) [138]. The cytoplasmic p53 participates in the negative regulation of autophagy based on its interaction with high mobility group box 1 (HMGB1) protein [139]. Importantly, in response to DNA damage cytoplasmic p53 may lead to apoptosis via activation of pro-apoptotic Bax, whereas nuclear p53 may induce expression of Bax and p53 upregulated modulator of apoptosis (PUMA), resulting in apoptosis [140].

It should be stressed that cytotoxic agents usually exert pleiotropic effects and their mechanisms of action involve multiple targets. Depending on the dose, concentration, and time of exposure, they may lead to different effects, i.e., different cell responses in different cell types. Importantly, cancer cells exhibit genomic instability and are devoid of normal control mechanisms. They act spontaneously using different pathways and seem to go beyond known scientific definitions of cell death subroutines. The interplay between autophagy and apoptosis in cancer cells is not yet fully understood and deserves further studies. It should be considered in anti-cancer therapy strategies, especially therapies based on combination treatment. It is important to elucidate the role of autophagy in different cancer types following drug treatment.

## 6. Autophagy and Apoptosis in the Mechanisms of Action of Stilbenes in Cancer Cells

### 6.1. RES

RES has been reported to have anti-oxidative, anti-microbial (anti-bacterial and anti-viral), anti-fungal, anti-diabetic, neuroprotective, cardioprotective, anti-inflammatory, and estrogenic activities [141,142,143,144,145,146,147,148,149,150]. Numerous in vitro studies have shown that RES exhibits anti-proliferative activity and is able to induce apoptosis in various human cancer cell lines including lung, oral, esophageal, gastric, colon, liver, pancreatic, breast, ovarian, and prostate cancer, as well as melanoma, leukemia, lymphoma, and multiple myeloma cell lines [151,152,153,154,155,156,157,158,159,160,161,162,163,164]. Accumulating evidence suggests that RES is able to induce both apoptosis and autophagy in lung, oral, esophageal, colon, breast, ovarian, cervical, endometrial, renal cancer cell lines, and glioblastoma, leukemia, and multiple myeloma cell lines [165,166,167,168,169,170,171,172,173,174,175,176,177,178,179,180,181,182,183,184,185,186]. Moreover, many in vivo studies have demonstrated the anti-cancer activity of RES [155,187,188,189,190].

Accumulating evidence suggests that RES possesses immunomodulatory activity and may be promising in supporting cancer immunotherapy [191,192,193]. Noteworthily, RES was reported to activate natural killer (NK) cells [192]. NK cells are able to eliminate cancer cells and play an important role in innate immune response [194]. RES increased interferon-gamma (IFN-ɣ) secretion, a cytokine important in cancer cell clearance. Moreover, RES was found to inhibit the proliferation of MDA-MB-231 breast cancer cells by promoting the M1/M2 macrophage polarization ratio and suppressing the interleukin 6/phosphorylated signal transducer and activator of transcription 3 (pSTAT3) pathway [193]. Tumor-associated macrophages (TAMs) represent an abundant leukocyte population in the tumor microenvironment [195,196]. Macrophages influenced by different factors may change their phenotype and differentiate into pro-inflammatory M1 or anti-inflammatory M2 phenotypes [196]. M1-like TAMs inhibit tumor growth, whereas M2-like TAMs promote tumor development [195].

#### 6.1.1. Lung Cancer

RES was reported to induce both autophagy and apoptosis in human non-small cell lung cancer (NSCLC) cells [166,167,168,169]. Lung cancers remain the leading cause of cancer-related deaths worldwide. About 80% to 85% of lung cancers are NSCLC [169]. There are several NSCLC subtypes such as adenocarcinoma, squamous cell carcinoma, and large cell carcinoma. Several studies focused on the in vitro effects of RES in the A549 human NSCLC cell line [166,167,168]. The relationship between autophagy and apoptosis appears to be dependent on RES concentration and the time of treatment of cells with this compound [165,197,198,199].

RES was found to inhibit the proliferation of A549 human lung adenocarcinoma cells in a dose-dependent manner [165]. Following the treatment of cells with 25, 100, and 200 µM RES, an increased percentage of apoptotic annexin V-positive cells was observed as revealed by the flow cytometry (Table 2). This effect was the most pronounced at a concentration of RES of 200 µM. Moreover, RES also induced autophagy in A549 cells (Table 2). RES treatment dose-dependently increased Beclin 1 protein level and LC3-II/LC3-I protein ratio, while decreasing the p62/SQSTM1 protein level. Of note, the p62/SQSTM1 protein is a selective autophagy receptor protein that interacts with ubiquitinated proteins and LC3-II [90,99]. The fluorescence microscopic examination of A549 cells transfected with GFP-LC3 plasmid and treated with 200 µM RES showed LC3-positive fluorescent puncta accumulation, indicative of the formation of autophagic vacuoles. Interestingly, RES appeared to induce autophagy in A549 cells by increasing the expression of silent information regulator 1 (SIRT1). SIRT1 is a NAD^+^-dependent deacetylase involved in gene expression regulation based on deacetylation of lysine residues on histones [200]. Noteworthily, RES was reported to have the ability to activate SIRT1 [200]. The co-treatment of cells with 200 µM RES and an autophagy inhibitor 3-methyladenine (3-MA) or with a SIRT1 inhibitor nicotinamide inhibited cell proliferation and promoted apoptosis compared with the treatment with RES alone, suggesting that RES-induced autophagy in A549 cells played a pro-survival role. 3-MA is an inhibitor of class III PI3K [201]. The class III PI3K is a member of the class III PI3K complex I that is recruited in the formation of the phagophore, i.e., in the nucleation step of autophagy [94]. Furthermore, RES was found to induce autophagy by inhibiting the Akt/mammalian (mechanistic) target of rapamycin (mTOR) and activating the p38-MAPK signaling pathway [165]. RES inhibited phosphorylation of Akt, mTOR, and p70 ribosomal protein S6 kinase (p70S6K), while increased phosphorylation of p38, leading to a decreased ratio of p-Akt/Akt, p-mTOR/mTOR, p-p70S6K/p70S6K and an increased ratio of p-p38/p38.

Other authors also showed that RES decreased the viability of A549 cells in a dose- and time-dependent manner [166]. The half-maximal inhibitory concentration IC_50_ estimated after treatment of A549 cells with RES for 48 and 72 h were 91.77 and 71.71 µM, respectively. RES at the concentration of 50 µM and higher increased the percentage of annexin V-positive or TUNEL-positive cells, indicative of apoptosis (Table 2). RES also led to caspase-3 cleavage. It decreased *BCL2* mRNA and Bcl-2 protein levels. Moreover, it increased *BAX* mRNA and Bax protein levels. Additionally, RES induced autophagy in A549 cells as revealed by the detection of autophagic vacuoles by the fluorescence microscopy following monodansylcadaverine (MDC) staining (Table 2). Transmission electron microscopy (TEM) showed an accumulation of autophagosomes. Some of them contained damaged mitochondria or endoplasmic reticulum. Treatment of A549 cells with 25, 50, and 100 µM RES decreased p62 protein level and increased Beclin 1 protein level as well as increased LC3-II/LC3-I protein ratio. In the presence of 3-MA, the viability of RES-treated cells was higher than in the absence of 3-MA, suggesting the pro-death role of autophagy in RES-treated cells. The authors of the study also found that RES modulated apoptosis and autophagy in A549 cells via a p53-dependent signaling pathway [166].

Another study also demonstrated that RES inhibited cell growth and induced autophagy and apoptosis in A549 cells (Table 2) [167]. RES treatment resulted in a dose-dependent increase in LC3-II protein level and accumulation of LC3-positive fluorescent puncta, indicative of autophagy. Moreover, RES induced apoptosis in A549 cells. RES at the concentration of 300 µM led to PARP-1 cleavage, caspase-3 cleavage, phosphatidylserine externalization, loss of mitochondrial membrane potential, and cytochrome c release, as well as increased Bax and decreased Bcl-2 protein levels. Notably, apoptotic effects were negligible at RES concentrations lower than 300 µM, namely at 100 or 200 µM. In the presence of the pan-caspase inhibitor Z-VAD-FMK, the viability of RES-treated A549 cells was higher in comparison with RES treatment alone. RES induced non-canonical autophagy, Beclin 1 and ATG5-independent, since both Beclin 1 and ATG5 protein levels decreased following RES treatment. Silencing of *BECN1* and *ATG5* genes with siRNAs barely inhibited LC3 lipidation in A549 cells. Furthermore, RES was found to induce pro-survival mitophagy in A549 cells, protecting the cells from apoptosis [167].

A recent study also showed that RES was able to induce both autophagy and apoptosis in A549 cells (Table 2) [168]. It decreased the viability of A549 cells in a concentration- and time-dependent manner. The effects of RES on autophagy activation in A549 cells were concentration-dependent. The authors of the study found that 55 µM RES induced protective autophagy in A549 cells. However, autophagy induced by RES at a concentration higher than 55 µM played a pro-death, rather than protective role in A549 cells. Treatment of cells with 25, 50, and 75 µM RES resulted in increased Beclin 1 and LC3B protein levels as well as increased LC3-II/LC3-I protein ratio. Noteworthily, in the presence of 3-MA, *BECN1* mRNA and Beclin 1 protein levels decreased in RES-treated cells. Autophagic vacuoles were detected in RES-treated cells following MDC staining, as well as after transfection of cells with adenovirus mRFP-GFP-LC3. TEM micrographs showed autophagosomes, autolysosomes, and apoptotic cells. Accumulation of autophagic vacuoles in RES-treated A549 cells was also determined by the flow cytometry after acridine orange (AO) staining. Simultaneously, following treatment with 25, 50, and 75 µM RES the percentage of apoptotic cells increased as determined using annexin V-FITC/PI and TUNEL-FITC/PI assays. Additionally, increased *BAX* mRNA and Bax protein levels, as well as decreased *BCL2* mRNA and Bcl-2 protein levels, were detected. The pan-caspase inhibitor Z-VAD-FMK partially reversed RES-induced effects concerning Bax and Bcl-2. Notably, the percentage of apoptotic cells decreased in the presence of 3-MA in comparison with the treatment with RES alone. The flow cytometric analysis revealed that the inhibitory effect of Z-VAD-FMK on the accumulation of autophagic vacuoles in A549 cells depended on RES concentration. It was absent after treatment of cells with 30 µM RES and appeared following treatment with 80 µM RES. Furthermore, RES increased both nerve growth factor receptor (*NGFR*) mRNA level and NGFR protein level in A549 cells. RES-induced autophagy and apoptosis in A549 cells were found to be regulated by the NGFR-AMPK-mTOR signaling pathway [168].

RES was found to inhibit the growth of gefitinib-resistant human NSCLC cell line PC9/G (lung adenocarcinoma) [169]. Gefitinib is an epidermal growth factor receptor (EGFR) tyrosine kinase inhibitor used in NSCLC treatment [169]. It provides clinical benefits to NSCLC patients with EGFR mutations. However, the acquired resistance to gefitinib often leads to gefitinib treatment failure. The IC_50_ value for gefitinib in PC9/G cells was 6.36 μM, with a 302-fold increase relative to that in the gefitinib-sensitive PC9 cells (0.021 μM). RES induced autophagy, apoptosis, and cell senescence in PC9/G cells (Table 2). Treatment of cells with 40 µM RES for 72 h resulted in an increased LC3B-II protein level, indicative of autophagy. These results were corroborated by the fluorescence microscopic examination and the flow cytometric analysis after MDC staining that revealed formation of autophagic vacuoles. Induction of apoptosis was confirmed by the detection of condensation and fragmentation of cell nuclei by fluorescence microscopy following DAPI staining. In addition, annexin V-positive cells were detected by the flow cytometry. The Western blotting analysis showed increased protein levels of p53, p21, and cleaved caspase-3 after exposure of PC9/G cells to RES. Moreover, cell senescence characteristics, such as an increased proportion of SA-β-gal-positive cells, were observed. Furthermore, it was also found that combined gefitinib and RES treatment was able to overcome the acquired resistance of PC9/G cells to gefitinib [169].

#### 6.1.2. Oral Cancer

RES was reported to induce autophagy and apoptosis in cisplatin-resistant CAR human oral cancer cells (Table 2) [170]. The CAR cell line was developed by exposing the parental CAL 27 human tongue squamous cell carcinoma cell line to cisplatin. RES inhibited the proliferation of CAR cells in a concentration-dependent manner. The IC_50_ values of RES evaluated after 24, 48, and 72 h of treatment of CAR cells were 95.23, 73.23, and 51.62 µM, respectively. Importantly, the authors of the study also found that the IC_50_ value of RES in human gingival fibroblasts was over 100 µM. After treatment of CAR cells with 50 µM RES, autophagy-specific markers and accumulation of autophagic vacuoles were detected by fluorescence microscopy using an LC3B-GFP assay kit and several other methods, such as staining of cells with AO, LysoTracker Red DND-99, and MDC. RES increased protein levels of Beclin 1, LC3-II, ATG5, ATG7, ATG12, autophagy related 14 (ATG14), ATG16L1, class III PI3K, and decreased the protein level of Rubicon. Notably, in CAR cells treated with 50 µM RES, DNA condensation and apoptotic DNA breaks were detected by fluorescence microscopy following DAPI staining and TUNEL staining. RES treatment increased protein levels of cytochrome c, Apaf-1, AIF, endonuclease G (Endo G), Bax, Bad, cleaved caspase-3, and cleaved caspase-9. Moreover, decreased protein levels of Bcl-2 and phosphorylated Bad (p-Bad) were detected in RES-treated cells. The aforementioned autophagic and apoptotic effects were the most pronounced at RES concentrations of 50 and 100 µM. The viability of cells treated with RES in the presence of an autophagy inhibitor 3-MA, an AMPK inhibitor compound c, or a pan-caspase inhibitor Z-VAD-FMK was higher than the viability of cells treated with RES alone. Furthermore, RES was found to exert the effects related to autophagy and apoptosis in CAR cells by the modulation of Akt and AMPK signaling [170].

#### 6.1.3. Esophageal Cancer

RES reduced the cell viability and inhibited the colony formation of EC109 and EC9706 human esophageal squamous cell carcinoma [171]. IC_50_ values of RES determined following 24 h of treatment were 120 and 80 µM for EC109 and EC9706 cells, respectively. RES induced apoptosis in both studied cell lines as determined by the detection of chromatin condensation, phosphatidylserine externalization, caspase-3 activation, increased Bax protein level, and decreased Bcl-2 protein level (Table 2). In addition to apoptosis, RES also induced autophagy in EC109 and EC9706 cells (Table 2). It led to an increase in LC3-II, Beclin 1, and ATG5 protein levels. The accumulation of autophagic vacuoles was also detected by the immunofluorescence assay with anti-LC3 antibodies, as well as by MDC staining and AO staining. Pretreatment of EC109 and EC9706 cells with chloroquine (CQ) followed by treatment with 150 µM RES resulted in decreased Beclin 1 and increased LC3-II protein levels in comparison with the effects of RES alone. CQ is a lysosomal lumen alkalizer that inhibits autophagy by impairing lysosomal acidification and fusion of autophagosomes with lysosomes [201,202]. Pretreatment of EC109 and EC9706 cells with an autophagy inhibitor 3-MA inhibited RES-induced LC3 and Beclin 1 protein expression. Both autophagy inhibitors CQ and 3-MA enhanced RES-induced apoptotic cell death in both studied cell lines. Knockdown of *BECN1* and *ATG5* genes with siRNAs resulted in reduced LC3-II levels in RES-treated cells and enhanced apoptosis compared with untreated cells. Furthermore, it was found that RES-induced autophagy in EC109 and EC9706 cells was not mediated by the AMPK/mTOR pathway [171].

#### 6.1.4. Colon Cancer

RES was found to inhibit the growth of HT-29 human colon cancer cells in a dose- and time-dependent manner [172]. The IC_50_ value of RES calculated after treatment of HT-29 cells for 72 h was 115.9 µM. RES induced apoptosis in HT-29 cells, characterized by phosphatidylserine externalization, poly(ADP-ribose) polymerase (PARP) cleavage, and caspase-3,-8 cleavage (Table 2). RES also induced autophagy in HT-29 cells (Table 2). RES treatment led to an increase in LC3-II protein levels. TEM micrographs showed numerous autophagosomes in RES-treated cells. Additionally, confocal micrographs revealed an increased number of LC3-positive fluorescent puncta following RES treatment. In the presence of autophagy inhibitor 3-MA, the LC3-II protein level decreased in HT-29 cells compared with RES treatment alone. Moreover, in the presence of 3-MA, protein levels of cleaved caspase-3 and caspase-8 decreased and the percentage of annexin V-positive cells reduced compared with RES treatment alone. In the presence of the pan-caspase inhibitor Z-VAD(OMe)-FMK, the LC3-II protein level in RES-treated cells increased in comparison with RES treatment alone. Furthermore, RES induced oxidative stress in HT-29 cells. Incubation of cells with 150 µM RES in the presence of N-acetyl cysteine decreased the intracellular production of ROS, as well as the protein levels of LC3-II, cleaved caspase-3, and cleaved caspase-8, indicating that intracellular ROS may serve as an upstream stimulus controlling both autophagy and apoptosis in RES-treated HT-29 cells [172].

RES at the concentration of 100 µM was reported to induce pro-survival autophagy in DLD1 human colorectal cells that switched to caspase-dependent apoptosis (Table 2) [173]. Interestingly, the effect of RES related to the induction of autophagy was reversible on removal of the drug. The authors employed DLD1-transfected cells transiently expressing the chimeric fluorescent protein GFP–LC3 or Beclin 1–GFP and following RES treatment detected cytosolic redistribution of LC3 and Beclin 1. Additionally, the presence of autolysosomes was visualized by labeling the cells with MDC. Moreover, the colocalization of LC3 (a marker of autophagic vacuoles) and Lamp1 (a lysosomal marker) was also noted. The cytotoxic effect of RES in DLD1 cells was autophagy-dependent, as it was prevented either by downregulation of autophagy by asparagine (Asn) or RNA interference knockdown of *BECN1* as well as by expressing a dominant-negative form of Vps34 (the yeast homolog of class III PI3K) devoid of lipid kinase activity. In comparison with RES treatment alone, the percentage of annexin V-positive cells decreased after treatment of DLD1 cells with RES in the presence of Asn, Z-VAD-FMK, or both Asn and Z-VAD-FMK. Z-VAD-FMK failed to inhibit autophagy, whereas *BECN1* knockdown with specific siRNA abrogated the formation of autophagosomes as well as apoptosis in RES-treated cells. When DLD1 cells were infected with a recombinant adenoviral vector coding for a mutant Vps34 protein devoid of lipid kinase activity (Ad-Vps34dn), the expression of Vps34dn prevented the induction of autophagy and cell death. siRNA-mediated silencing of *LAMP2* prevented the formation of autolysosomes and decreased the percentage of annexin V-positive cells, suggesting that RES-induced autophagy-dependent cell death was due to the excessive accumulation of autolysosomes [173].

#### 6.1.5. Breast Cancer

RES was shown to induce pro-survival autophagy in MDA-MB231 breast cancer cells (Table 2) [174]. Treatment of cells with 120 µM RES led to an increase in LC3-I and LC3-II protein levels. Moreover, treatment with RES in the presence of 3-MA decreased LC3-II protein levels in comparison with the effects of RES alone. Additionally, RES induced caspase-3 cleavage, indicative of apoptosis (Table 2) and that effect was enhanced in the presence of 3-MA. The combination of RES and 3-MA reduced the viability of MDA-MB231 cells compared with RES treatment alone. Silencing of autophagy genes *ATG5* or *BECN1* by siRNA inhibited RES-induced LC3-II accumulation in MDA-MB231 cells. Moreover, knockdown of these genes resulted in increased caspase-3 activation in RES-treated cells as compared with the control. Furthermore, the authors showed that RES-mediated autophagy may involve Beclin 1 interaction with p53 in the cytosol and mitochondria [174].

#### 6.1.6. Ovarian Cancer

RES was found to be cytotoxic to A2780 human ovarian cancer cells [175]. Treatment of A2780 cells with 50 µM RES resulted in the accumulation of cells in the S-phase, whereas 100 µM RES caused the accumulation of cells in the G0/G1 phase of the cell cycle. RES-induced cell death was not blocked by the overexpression of Bcl-2 or Bcl-XL. RES induced apoptosome complex formation and caspase-9 cleavage in A2780 cells, indicative of apoptosis induction (Table 2). Moreover, RES caused the release of cytochrome c from mitochondria and that effect was partially inhibited by the pan-caspase inhibitor Z-VAD-FMK. TUNEL-positive cells were also detected following RES exposure. Fluorescence micrographs following Hoechst 33258 staining did not show apoptotic changes in nuclei in RES-treated A2780 cells, whereas MDC staining revealed an accumulation of autophagic vacuoles (Table 2). TEM micrographs revealed an accumulation of autophagic vacuoles in RES-treated cells, as well as an intact nuclear membrane and an absence of chromatin condensation.

RES was reported to induce autophagy and apoptotic cell death in OVCAR-3 human ovarian cancer cells (Table 2) [176]. Treatment of OVCAR-3 cells with RES resulted in a dose-dependent decrease in cell viability. Incubation of cells with 30 or 100 µM RES increased the percentage of annexin V-positive cells. RES also induced oxidative stress, decreased mitochondrial membrane potential, and increased caspase-3 and PARP activity. Following exposure of OVCAR-3 cells to 100 µM RES, protein levels of ATG5, LC3-II, and p62 increased. Inhibition of autophagy with an autophagy inhibitor CQ attenuated RES-induced apoptosis in OVCAR-3 cells. Similarly, inhibition of autophagy by the knockdown of *ATG5* expression with *ATG5* siRNA ameliorated RES-induced apoptotic cell death in OVCAR-3 cells. Importantly, cells treated with *ATG5* siRNA in combination with Z-VAD-FMK were more protected from RES-induced cell death compared with the cells treated with either inhibitor alone. A time course study revealed that autophagy was activated earlier than apoptosis, suggesting that autophagy was upstream of apoptosis in RES-treated OVCAR-3 cells [176].

#### 6.1.7. Cervical Cancer

Treatment of HeLa human cervical cancer cells with RES resulted in cell growth inhibition [177]. Exposure of cells to 100 µM RES induced phosphatidylserine externalization, caspase-3 cleavage, and cytochrome c release, indicative of apoptosis (Table 2). Moreover, treatment with 100 µM RES resulted in an autophagic response in HeLa cells (Table 2). The formation of autophagic vacuoles was determined by TEM as well as by fluorescence microscopy following MDC staining. Moreover, transfection of cells with pGFP-LC3 with the subsequent fluorescence microscopy examination showed GFP-LC3-positive fluorescent puncta, indicative of autophagy. The immunoblotting analysis showed the conversion of LC3-I to LC3-II. In the presence of protease inhibitors, E64 and PSTA, an increased LC3-II protein level was noted in RES-treated HeLa cells. Notably, the protein expression level of cathepsin L in RES-treated cells increased in time, whereas cathepsin B and D protein levels remained unchanged. Moreover, in the presence of E64 (an inhibitor specific for cathepsin B and L) and cathepsin L inhibitor I, the percentage of annexin V-positive cells increased in comparison with RES treatment alone. Furthermore, cathepsin L appeared to be an essential mediator of RSV-induced apoptosis in HeLa cells. Importantly, inhibition of autophagy with wortmannin or asparagine decreased the percentage of annexin V-positive cells in contrast to the treatment with RES alone [177].

#### 6.1.8. Endometrial Cancer

RES was found to inhibit the proliferation of Ishikawa endometrial cancer cells in a dose-dependent manner [178]. The IC_50_ value of RES was 20 µM. The cell cycle analysis demonstrated that 25 µM RES caused an increase in the sub-G1 cell population, indicative of apoptosis (Table 2). Moreover, an increased percentage of annexin V-positive cells was detected following RES treatment. RES also led to PARP cleavage. Exposure of Ishikawa cells to 25 and 100 µM RES increased the protein level of LC3-II, indicative of autophagy (Table 2). Confocal micrographs of RES-treated cells showed an accumulation of LC3-positive fluorescent puncta corresponding to autophagic vacuoles. Importantly, in the presence of the autophagy inhibitor CQ, the viability of cells decreased and the level of cleaved PARP as well as the percentage of annexin V-positive cells increased in comparison with RES treatment alone. Furthermore, autophagy inhibition by knockdown of *ATG5* and *ATG7* genes with specific siRNAs augmented RES-induced apoptosis in Ishikawa cells. The MTT assay revealed that the cells were more sensitive to RES when either *ATG5* or *ATG7* was silenced. Additionally, following RES treatment, the immunoblotting analysis revealed increased protein levels of p-AMPKα and phosphorylated extracellular signal-regulated kinase (p-ERK) and decreased protein level of p-AKT protein, suggesting that RES activated AMPK and ERK signaling in Ishikawa cells. The authors of the study also found that neither 25 µM nor 100 µM RES increased SIRT1 protein level. Taken together, RES-induced autophagy in Ishikawa cells appeared to play a pro-survival role [178].

#### 6.1.9. Renal Cancer

RES was reported to reduce the viability of 786-O human renal cell carcinoma in a time- and dose-dependent manner [179]. Renal cell carcinoma (RCC) is a subtype of kidney cancer. RES at the concentration of 40 µM induced apoptosis in 786-O cells as evidenced by phosphatidylserine externalization, disruption of mitochondrial membrane potential, caspase-3 activation, and PARP cleavage (Table 2). The pan-caspase inhibitor Z-VAD-FMK partially inhibited RES-induced apoptosis. In the presence of Z-VAD-FMK, the percentage of annexin V-positive cells was lower than in its absence. RES induced autophagy in 786-O cells (Table 2). Treatment of cells with 40 µM RES led to increased LC3-II protein level and accumulation of autophagic vacuoles. Inhibition of autophagy with autophagy inhibitor CQ or knockdown of *BECN1* gene with *BECN1* siRNA enhanced RES-induced apoptosis in 786-O cells. RES had no effect on p-AMPK and p-S6 protein levels, indicating that the AMPK-mTOR signaling pathway was not involved in RES-induced autophagy. Further experiments concerning the MAPK family-associated proteins (ERK, c-Jun NH(2)-terminal kinase (JNK) and p38) revealed that RES inhibited ERK, and activated both JNK and p38 [179].

#### 6.1.10. Glioblastoma

RES inhibited the growth of U251 human glioma cells in a time- and dose-dependent manner [180]. The flow cytometric analysis revealed that treatment of U251 cells with 150 µM RES led to an increase in the sub-G1 population and decreased mitochondrial membrane potential. Fluorescence micrographs following Hoechst 33258 staining showed other apoptotic characteristics such as chromatin condensation and nuclear fragmentation in RES-treated U251 cells (Table 2). Z-VAD-FMK partially inhibited RES-induced cell death. After treatment of U251 cells with 150 µM RES, an accumulation of MDC-positive as well as LC3-positive fluorescent puncta was detected, indicative of autophagic vacuoles (Table 2). Moreover, compared with untreated cells, in RES-treated cells, the protein levels of LC3-II and Beclin 1 increased. Furthermore, RES-induced autophagy appeared to play a cytoprotective role, since autophagy inhibitors 3-MA or Bafilomycin A1 (Baf A1) were able to partially diminish the cytotoxic effects of RES in U251 cells. Noteworthily, Baf A1 is a vacuolar-type H^+^-ATPase inhibitor that inhibits the acidification of lysosomes and prevents the maturation of autophagic vacuoles [201].

Interestingly, RES at the concentration of 10 and 20 µM was demonstrated to significantly potentiate the cytotoxicity of temozolomide against U87MG, GBM8401, and GBM-SKH human glioma cells by suppressing temozolomide-induced pro-survival autophagy and increasing apoptotic cell death [203]. Of note, temozolomide is a chemotherapeutic drug clinically used in the treatment of malignant gliomas [204].

#### 6.1.11. Leukemia

RES was shown to induce apoptosis and autophagy in K562 human myelogenous leukemia cells (Table 2) [181]. RES reduced the viability of K562 cells in a concentration- and time-dependent manner. It induced apoptosis in K562 cells that was associated with phosphatidylserine externalization and increased caspase-3 activity. RES at the concentration of 50 and 100 µM also induced autophagy in K562 cells as revealed by TEM micrographs showing accumulation of autophagic vacuoles. Moreover, an increased LC3-II protein level was detected. Treatment of K562 cells with 100 µM RES increased mRNA expression levels of several genes encoding proteins such as glycophorin A, HBA1, HBB, and γ-globin, indicating that the compound is able to induce erythroid differentiation in K562 cells. These results were corroborated by the flow cytometry analysis that revealed an increased protein expression of glycophorin A, CD71 antigen, and Band3 protein characteristic for the erythropoiesis lineage cell differentiation [181].

RES was demonstrated to induce autophagy and apoptosis in imatinib-sensitive (IM-S) and imatinib-resistant (IM-R) K562 cells (Table 2) [182]. Treatment of IM-S and IM-R K562 cells with 50 µM RES led to the accumulation of autophagic vacuoles as revealed by TEM. RES also increased LC3-II and p62 protein levels. Treatment of IM-S and IM-R K562 cells with RES in the presence of an autophagy inhibitor Baf A1 or the pan-caspase inhibitor Z-VAD-FMK increased the cell viability in comparison with the treatment with RES alone. Moreover, inhibition of caspase activation had no effect on RES-mediated autophagy, whereas inhibition of autophagy inhibited RES-induced caspase activation and apoptosis. Thus, RES-mediated autophagy contributed to RES-induced apoptosis. Moreover, RES triggered JNK and AMPK activation. It regulated autophagy through AMPK-dependent inhibition of the mTOR pathway. It increased AMPK phosphorylation on threonine 172 in both IM-S and IM-R K562 cells, which was accompanied by decreased phosphorylation of mTOR, p70-S6 kinase, S6 ribosomal protein, and eukaryotic initiation factor 4E-binding protein 1 (4EBP1). RES-mediated JNK activation resulted in an increased mRNA expression level of the *SQSTM1* gene and an increased p62/SQSTM1 protein level. The authors found that blockage of autophagy by siRNAs targeting genes encoding LC3, p62/SQSTM1, or AMPK proteins promoted survival of IM-S and IM-R cells, indicating that RES induced autophagic cell death [182].

The induction of both autophagy and apoptosis was also demonstrated in RES-treated Adriamycin-resistant K562 cells (K562/ADM) (Table 2) [183]. The IC_50_ value of RES in K562/ADM cells after 48 h of treatment with the compound was 57.7 µM. TEM revealed that RES at the concentration of 40 or 80 µM led to the accumulation of autophagic vacuoles. Furthermore, the number of MDC-positive fluorescent puncta was markedly greater compared with control cells. Following treatment of K562/ADM cells with RES, LC3-II, and Beclin 1 protein levels increased and p62 protein levels decreased, indicating induction of autophagy. RES treatment increased the protein levels of cleaved caspase-3 and Bax and decreased the protein level of Bcl-2. Moreover, the protein level of cathepsin D increased, suggesting that this lysosomal protease may be involved in the switch between autophagy and apoptosis in RES-treated K562/ADM cells. Noteworthily, pretreatment of cells with 3-MA prior to RES exposure resulted in increased cell viability and a decreased percentage of annexin V-positive cells in comparison with RES treatment alone. Thus, apoptosis induced by RES in K562/ADM cells was autophagy-dependent [183].

Our studies demonstrated that RES modulated autophagy and induced apoptosis in MOLT-4 human lymphoblastic leukemia and HL-60 human promyelocytic leukemia cells (Table 2) [184]. Treatment of MOLT-4 cells with 41 µM (IC_90_) RES for 24 and 48 h increased LC3-II and p62/SQSTM1 protein levels, suggesting inhibition of autophagy. Incubation of HL-60 cells with 43 µM (IC_90_) RES for 24, 48, and 72 h resulted in an increase in both LC3-I and LC3-II protein levels. However, p62/SQSTM1 level in HL-60 cells increased after 24, but decreased after 48 and 72 h of RES exposure. Importantly, the immunofluorescent staining with anti-LC3 antibodies revealed an accumulation of LC3-positive fluorescent puncta in RES-treated MOLT-4 and HL-60 cells, indicative of an accumulation of autophagic vacuoles. Moreover, RES induced apoptotic cell death in both studied cell lines, characterized by phosphatidylserine externalization, disruption of the mitochondrial membrane potential, caspase-3 activation, internucleosomal DNA fragmentation, PARP1 cleavage as well as morphological changes such as chromatin condensation and fragmentation of cell nuclei [184].

Another study also showed that RES triggered autophagy and apoptosis in HL-60 cells (Table 2) [185]. RES-induced apoptosis in HL-60 cells occurred via both intrinsic and extrinsic apoptotic pathways, which was corroborated by the detection of caspase-3 activity, phosphatidylserine externalization, decreased mitochondrial membrane potential, increased Bax/Bcl-2 ratio, and increased Fas and FasL protein levels, as well as characteristics for apoptosis cleavage of caspase-3, caspase-8 and Bid. The combined treatment of HL-60 cells with RES and caspase-3 inhibitor Z-DEVD-FMK increased the cell viability in comparison with RES treatment alone. RES triggered autophagy in HL-60 cells. LC3-II, ATG5, and Beclin 1, as well as p62 protein levels, increased after RES exposure. The immunofluorescence analysis showed an accumulation of LC3-positive fluorescent puncta in RES-treated HL-60 cells. The co-treatment with RES and 3-MA increased the cell viability and decreased the caspase-3 activity compared with RES treatment alone. Furthermore, RES was found to induce apoptotic cell death in HL-60 cells which was dependent on autophagy activated through the PI3K-AKT and liver kinase B1 (LKB1)-AMPK-regulated mTOR signaling pathways [185].

Noteworthily, RES in combination with arsenic trioxide (As_2_O_3_) has been reported to exert synergistic anti-leukemic activity [205]. Co-treatment of NB4 human promyelocytic leukemia cells with 5 µM RES and 2 µM arsenic trioxide resulted in oxidative stress, decreased superoxide dismutase activity, mitochondrial damage, apoptosis, and autophagy. Moreover, RES was also found to alleviate arsenic trioxide-induced cardiotoxicity.

#### 6.1.12. Multiple Myeloma

RES was reported to reduce the viability of U266, RPMI-8226, and NCI-H929 multiple myeloma cells in a time- and dose-dependent manner [186]. It markedly inhibited the colony formation in these cell lines. RES at the concentration of 50 and 100 µM induced apoptosis in U266, RPMI-8226, and NCI-H929 cells characterized by phosphatidylserine externalization, a decrease in Survivin protein level, cleavage of caspase-3 and PARP (Table 2). The TUNEL assay also corroborated the induction of apoptosis in the studied cell lines. In addition, incubation of cells with 50 and 100 µM RES led to an increase in both LC3-II and Beclin 1 protein levels (Table 2). Noteworthily, treatment with RES in the presence of 3-MA partially increased the cell viability and decreased the percentage of apoptotic U266, RPMI-8226, and NCI-H929 cells, indicating that apoptosis was partially dependent on autophagy. RES increased phosphorylation of AMPKα (Thr172) and decreased phosphorylation of mTOR (Ser2448), p70S6K (Thr389), and 4EBP1 (Thr37/46) in U266, RPMI-8226, and NCI-H929 cells, suggesting that AMPK and mTOR pathways may be involved in RES-induced autophagy [186].

### 6.2. PTER

PTER has attracted researchers’ attention due to its anti-oxidative, anti-bacterial, anti-viral, anti-inflammatory, anti-diabetic, neuroprotective, cardioprotective, and anti-cancer activities [206,207,208,209,210,211,212]. Numerous in vitro studies have demonstrated that PTER is able to inhibit growth and induce apoptosis in many human cancer cell types including lung, gastric, pancreatic, liver, colon, breast, and prostate cancer as well as osteosarcoma and leukemia cells [213,214,215,216,217,218,219,220]. Recently, it has also been shown to induce both apoptosis and autophagy in human lung, oral, colon, pancreatic, breast, and bladder cancer and leukemia cell lines [221,222,223,224,225,226,227,228]. In vivo studies also confirmed the anti-cancer properties of PTER [229,230,231,232]. Importantly, PTER exhibits greater bioavailability than RES [233].

#### 6.2.1. Lung Cancer

PTER inhibited cell growth and induced cell cycle arrest, apoptosis, and autophagy in both chemosensitive and chemoresistant A549 human lung cancer cells (Table 2) [221]. In this study docetaxel-induced multidrug-resistant cell subline A549/D16 was established. The flow cytometric analysis following annexin V/PI staining revealed phosphatidylserine externalization in both chemosensitive A549 and chemoresistant A549/D16 cells. Fluorescence micrographs following DAPI staining showed other apoptotic characteristics such as condensed and fragmented nuclei. In A549 and A549/D16 cells transfected with pEGFPC1-LC3 after treatment with PTER an increase in GFP-LC3 fluorescent puncta was detected. Following PTER treatment, LC3-II and Beclin 1 protein levels increased, whereas the p62/SQSTM1 protein level decreased in both studied cell lines. Moreover, in PTER-treated A549 and A549/D16 cells a concentration- and time-dependent formation of acidic vesicular organelles (AVOs) was detected as determined by the flow cytometry after AO staining. The aforementioned effects were the most prominent at the concentration of PTER of 100 µM. Compared with A549 and A549/D16 cells treated with 100 µM PTER alone, in the presence of autophagy inhibitors 3-MA or Baf A1, the formation of AVOs was significantly reduced, whereas the percentage of annexin V-positive cells significantly increased. Similarly, transfection with *BECN1* siRNA followed by PTER treatment resulted in an increased percentage of annexin V-positive cells compared with the control. Thus, inhibition of autophagy in A549 and A549/D16 cells enhanced the cytotoxicity of PTER. Notably, in the presence of the broad-spectrum caspase inhibitor Z-VAD-FMK, the percentage of annexin V-positive cells decreased, whereas the percentage of cells with AVOs remained unaltered in comparison with cells treated with PTER in the absence of Z-VAD-FMK. Furthermore, after treatment of A549 and A549/D16 cells with 100 µM PTER protein levels of phosphorylated PI3K (p-PI3K), Akt (p-Akt), and JNK (p-JNK) decreased, whereas the protein level of phosphorylated ERK1/2 (p-ERK1/2) increased. These findings indicated that PTER-induced autophagy was mediated by the inhibition of PI3K/AKT and JNK pathways and activation of the ERK1/2 pathway [221].

#### 6.2.2. Oral Cancer

PTER was reported to inhibit cell growth, cause cell cycle arrest, and induce both autophagy and apoptosis in SAS and OECM-1 human oral cancer cells (Table 2) [222]. After transfection of cells with pEGFPC1-LC3, followed by PTER treatment, an increase in GFP-LC3 fluorescent puncta was observed. The flow cytometric analysis after AO staining revealed that treatment of SAS and OECM-1 cells with PTER for 24 h led to the accumulation of AVOs in a concentration- and time-dependent manner. Compared with the control, after 24 h of treatment with 10, 20, or 40 µM PTER, protein levels of LC3-II and Beclin 1 increased in both cell lines. Treatment of SAS and OECM-1cells with 40 µM PTER in the presence of 3-MA or Baf A1 resulted in increased cell viability in comparison with PTER treatment alone. PTER-treated SAS and OECM-1 cells showed apoptotic characteristics such as condensed and fragmented nuclei following DAPI staining. After treatment with PTER, the protein levels of cleaved PARP as well as cleaved caspase-3, -8, -9 increased in both cell lines. In comparison with the treatment of SAS and OECM-1 cells with PTER alone, in the presence of caspase-3, -8, -9-specific inhibitors, the viability of PTER-treated SAS and OECM-1 cells increased. In addition, in both cell lines, an increase in the percentage of annexin V-positive cells was detected. There were no significant changes between the percentage of annexin V-positive SAS and OECM-1 cells concerning the treatment of these cell lines in the presence or absence of autophagy inhibitors 3-MA and Baf A1. Treatment of SAS and OECM-1 cells with PTER in the presence of rapamycin, an mTOR inhibitor, decreased the cell viability, but the percentage of annexin V-positive cells was not significantly affected compared with PTER treatment alone. Moreover, PTER decreased levels of phosphorylated p38, AKT, extracellular signal-regulated kinase 1/2 (ERK1/2), mTOR, uncoordinated-51 (unc-51)-like kinase (ULK; Ser757) and increased levels of phosphorylated c-Jun NH_2_-terminal kinase 1/2 (JNK1/2), AMPK, regulatory-associated protein of mTOR (Raptor), ULK (Ser555) in both cell lines. Thus, PTER exerted its effects in SAS and OECM-1 cells by the inhibition of AKT, p38, ERK1/2, and the activation of JNK1/2 [222].

PTER was demonstrated to induce both autophagy and apoptosis in cisplatin-resistant CAR human oral cancer cells (Table 2) [223]. The IC_50_ values of PTER in CAR cells estimated after 24, 48, and 72 h of incubation were 78.26, 48.04, and 20.65 µM, respectively. PTER treatment led to the concentration-dependent accumulation of AVOs as revealed by the microscopic examination of cells following AO staining. The formation of autophagic vacuoles was also detected using other methods including MDC staining, LysoTracker Red staining, and Magic Red Cathepsin B assay kit. PTER also upregulated the protein expression of ATG5, ATG7, ATG12, Beclin 1, and LC3-II in CAR cells. 3-MA and CQ inhibited the accumulation of AVOs in PTER-treated CAR cells. Moreover, in the presence of these autophagy inhibitors, the viability of cells following PTER exposure was higher compared with PTER treatment alone, indicating that autophagy contributed to cell death. Furthermore, PTER induced apoptotic cell death in CAR cells via an intrinsic pathway. PTER treatment upregulated protein levels of Bax, cytochrome c, cleaved PARP, and cleaved caspases -9,-3,-7, as well as downregulated Bcl-2 protein level. In addition, an increased number of TUNEL-positive cells and activation of caspases -3,-9 were noted. Moreover, the microscopic examination following Hoechst 33342 staining revealed condensation of cell nuclei in PTER-treated cells. Notably, Z-VAD-FMK decreased the cytotoxicity of PTER to CAR cells. Interestingly, PTER significantly decreased mRNA expression of the *ABCB1* gene encoding multidrug resistance protein 1 (MDR1, P-glycoprotein, ABCB1) as well as decreased MDR1 protein level. Moreover, it decreased the protein level of phosphorylated AKT (p-AKT). Taken together, PTER was found to trigger autophagy and apoptosis in CAR cells by MDR1 suppressing and AKT signaling [223].

#### 6.2.3. Colon Cancer

PTER was found to inhibit cell growth and induce the G0/G1 cell cycle arrest and cell death in HT-29 human colon cancer cells [224]. Treatment of HT-29 cells with PTER led to caspase-3 activation, oligonucleosomal DNA fragmentation, and increased *BAX* mRNA expression level, indicating induction of apoptosis (Table 2). Moreover, other apoptotic characteristics such as an increased percentage of cells of the sub-G1 fraction were also noted. Importantly, PTER modulated autophagic pathways in HT-29 cells (Table 2). Following treatment with 60 µM PTER a significant increase in mRNA levels of autophagy-related genes *ULK1*, *AMBRA1,* and *MAP1LC3A* was observed. However, PTER did not affect the expression of a gene encoding the Beclin 1 protein. Furthermore, the downregulation of AKT and STAT3 pathways appeared to be involved in the mechanisms of action of PTER in HT-29 cells. Treatment of cells with 60 µM PTER decreased the phosphorylated-AKT/total AKT ratio. Exposure of cells to 10, 40, or 60 µM PTER resulted in a significant decrease in phosphorylated-STAT3/total STAT3 ratio.

#### 6.2.4. Pancreatic Cancer

PTER was shown to trigger autophagy and apoptosis in human pancreatic ductal adenocarcinoma BxPC-3 and MIA PaCa-2 cells (Table 2) [225]. Treatment of BxPC-3 and MIA PaCa-2 cells with 100 µM PTER led to the formation of AVOs. In addition, an increase in LC3-II, Beclin-1, and p62/SQSTM1 protein levels was detected. Simultaneously, apoptotic characteristics were noted in both cell lines following PTER treatment such as an increased percentage of annexin V-positive cells, as well as an increased percentage of cells of the sub-G0/G1 fraction. Moreover, co-treatment of BxPC-3 and MIA PaCa-2 cells with PTER and the autophagy inhibitor CQ resulted in inhibition of autophagy. Importantly, via inhibition of autophagy, CQ sensitized these cells to PTER-induced apoptosis. The co-treatment of BxPC-3 and MIA PaCa-2 cells with 100 µM PTER and 10 µM CQ exerted enhanced cytotoxicity against both studied cell lines compared with the treatment with PTER or CQ alone. Moreover, the combination of PTER and CQ decreased Bcl-XL and Bcl-2 protein levels and increased Bax and cleaved caspase-3 protein levels compared with the treatment with PTER or CQ alone. Thus, autophagy observed in PTER-treated BxPC-3 and MIA PaCa-2 cells played a pro-survival role. Furthermore, the combination of PTER with CQ appeared to act via downregulation of RAGE (receptor for advanced glycation end products)/STAT3 and AKT/mTOR pathways. The authors of the study also investigated the utility of this combination treatment in vivo using an orthotopic animal model and found that it significantly inhibited pancreatic cancer growth, delayed tumor quadrupling times, and inhibited autophagy and STAT3 in pancreatic tumors [225].

#### 6.2.5. Breast Cancer

Wang et al. showed that PTER inhibited cell growth and induced the G0/G1 cell cycle arrest in MCF-7 and Bcap-37 human breast cancer cells [226]. PTER at the concentration of 50 and 100 µM induced apoptotic cell death in both studied cell lines as shown by the detection of chromatin condensation, phosphatidylserine externalization, and PARP cleavage (Table 2). Compared with the control, following PTER treatment the LC3-II protein level markedly increased in both cell lines (Table 2). Furthermore, PTER was found to induce cytoprotective autophagy in MCF-7 and Bcap-37 cells [226].

#### 6.2.6. Bladder Cancer

PTER was found to induce both autophagy and apoptosis in sensitive T24 and nicotine-induced chemoresistant T24R human bladder cancer cells (Table 2) [227]. The IC_50_ value calculated after 48 h of PTER treatment was 66.58 and 77.95 µM for T24 cells and T24R cells, respectively. PTER at the concentration of 100 µM induced the G0/G1 cell cycle arrest that shifted to the S-phase arrest after 48 and 72 h of incubation. Following treatment with this polyphenol the percentage of cells in the sub-G0/G1 fraction increased in both studied cell lines in a concentration- and time-dependent manner. PTER-treated T24 and T24R cells showed apoptotic characteristics such as phosphatidylserine externalization, caspase-3 activation, chromatin condensation, and nuclear fragmentation. It also decreased protein levels of anti-apoptotic proteins Bcl-2 and Bcl-XL. Moreover, TEM micrographs showed an accumulation of autophagic vacuoles in PTER-treated cells. AO staining revealed the concentration- and time-dependent formation of AVOs in PTER-treated T24 and T24R cells. PTER led to the conversion of LC3-I to LC3-II. Noteworthily, after PTER treatment in the presence of 3-MA or Baf A1, the percentage of cells with AVOs significantly reduced compared with PTER treatment alone. Similar results were obtained by transfection of cells with *BECN1* shRNA. Pretreatment with 3-MA or transfection with *BECN1* shRNA decreased LC3-II protein level in PTER-treated T24 and T24R cells, whereas Baf A1 increased the level of this protein. Moreover, PTER treatment in the presence of 3-MA, Baf A1, or following *BECN1* shRNA transfection increased the percentage of annexin V-positive cells compared with PTER treatment alone. Pretreatment of cells with Z-VAD-FMK followed by PTER treatment reduced the percentage of annexin V-positive cells. The MTT assay revealed that in comparison with the treatment of T24 and T24R cells with PTER alone, the combination of PTER with 3-MA or Baf A1 decreased, whereas the combination of PTER with Z-VAD-FMK increased the cell viability. Furthermore, PTER was found to induce autophagy through inhibition of the AKT/mTOR/p70S6K pathway and activation of the MEK/ERK1/2 pathway in T24 cells [227].

#### 6.2.7. Leukemia

Our results showed that PTER modulated autophagy in HL-60 human promyelocytic leukemia cells (Table 2) [228]. It inhibited the growth of HL-60 cells and induced an accumulation of autophagic vacuoles followed by cell death. The IC_90_ value estimated after 72 h of incubation with PTER was 43 µM. Treatment with 43 µM PTER led to the formation of cytoplasmic vacuoles as revealed by the confocal microscopy after acridine orange/ethidium bromide staining. The vacuoles tend to enlarge in time. 3-MA prevented the formation of these vacuoles. Noteworthily, almost no vacuoles were visible following exposure to 1, 10, or 100 µM PTER. The immunofluorescence analysis revealed that vacuoles observed in HL-60 cells treated with 43 µM PTER were LC3-positive, indicating autophagy modulation. TEM micrographs corroborated these findings. In addition, 43 µM PTER led to the conversion of LC3-I to LC3-II. Moreover, PTER caused the G0/G1 cell cycle arrest (Table 2). PTER induced apoptosis in HL-60 cells, as shown by phosphatidylserine externalization, internucleosomal DNA fragmentation, caspase activation, and disruption of mitochondrial membrane potential. The caspase-8 inhibitor Z-IETD-FMK failed to block the PTER-induced apoptosis of HL-60 cells. However, the caspase-9 inhibitor Z-LEHD-FMK partially inhibited apoptosis, indicating the activation of an intrinsic (mitochondrial) apoptotic pathway.

### 6.3. PIC

PIC, a structural analog of resveratrol, has been demonstrated to exhibit a wide range of biological properties including anti-oxidative, anti-microbial, anti-diabetic, neuroprotective, cardioprotective, anti-inflammatory, and estrogenic activities [85,234,235,236,237,238,239,240]. Moreover, it has been reported to exert anti-proliferative and proapoptotic effects in many human cancer cell lines including pancreatic, colon, breast, prostate, and bladder cancer, as well as osteosarcoma, melanoma, leukemia, lymphoma, and multiple myeloma [241,242,243,244,245,246,247,248,249,250,251,252,253,254,255,256]. PIC has been reported to induce both apoptosis and autophagy in human neuroblastoma and leukemia cell lines [257,258]. Anti-cancer activity of PIC has also been demonstrated in in vivo studies employing animal models [190,259,260,261]. Of note, RES has been found to be converted to PIC by human cytochrome P450 enzyme CYP1B1 [262].

#### 6.3.1. Neuroblastoma

PIC induced caspase-dependent apoptosis and enhanced autophagy in SH-SY5Y human neuroblastoma cells (Table 2) [257]. It inhibited the viability of SH-SY5Y cells and the IC_50_ value evaluated after 72 h of treatment with the compound was 139.03 µM. Treatment of SH-SY5Y cells with 50 and 100 µM PIC for 72 h resulted in reduced colony formation and decreased cell invasion capacity. PIC-induced apoptosis in SH-SY5Y cells was characterized by phosphatidylserine externalization, decreased mitochondrial membrane potential, and DNA fragmentation. PIC significantly increased mRNA expression levels of *CASP3*, *CASP8*, *CASP9*, *BAX*, and *FADD* genes, indicative of the activation of intrinsic and extrinsic apoptotic signaling pathways. PIC also enhanced autophagic activity in SH-SY5Y cells as revealed by the detection of the increased number of autophagic structures as well as increased mRNA expression levels of *BECN1*, *ATG5*, *ATG7*, *ATG12*, *MAP 1LC3A*, and *MAP 1LC3B* genes.

#### 6.3.2. Leukemia

Our study demonstrated that PIC induced autophagy and apoptosis in MOLT-4 human acute lymphoblastic leukemia cells (Table 2) [258]. MOLT-4 cells were treated with PIC at the concentration of 45,5 µM, corresponding to the IC_90_ value. PIC activated autophagic pathways as revealed by the detection of an increased level of LC3-II protein and a concomitant decrease in p62/SQSTM1 protein level. The immunofluorescence analysis showed the accumulation of LC3-positive fluorescent puncta, indicative of autophagic vacuoles. In addition, PIC induced apoptotic cell death in MOLT-4 cells, documented by phosphatidylserine externalization, caspase-3 activation, and disruption of mitochondrial membrane potential. Treatment with PIC increased the number of cells in the sub-G1 fraction, suggesting apoptotic DNA degradation. Hoechst 33342 staining revealed chromatin condensation and fragmentation of cell nuclei. Moreover, other apoptotic characteristics were also detected such as PARP1 cleavage and internucleosomal DNA fragmentation. Noteworthily, MOLT-4 cells appeared to be able to acquire resistance to PIC toxicity since the toxic effects exerted by this compound diminished after longer periods of exposure. The detection of P-glycoprotein (P-gp, MDR1) activity and increased cell surface expression of this ATP-binding cassette (ABC) transporter after PIC treatment indicated that P-gp-positive cells survived and reconstituted the cell population. Moreover, PIC treatment resulted in decreased cell surface expression of another ABC transporter, namely breast cancer resistance protein (BCRP) in MOLT-4 cells. These findings suggest that MOLT-4 cells expressing BCRP are sensitive to PIC toxicity. Importantly, we obtained similar results using different leukemia cell line. Our study showed that HL-60 human acute promyelocytic leukemia cells are also able to acquire resistance to PIC toxicity via mechanisms involving ABC transporters [263].

### 6.4. OXYRES

OXYRES has been found to have anti-oxidant, anti-microbial, anti-inflammatory, neuroprotective, hepatoprotective, and anti-cancer activities [264]. It exhibited cytotoxicity against A2780 human ovarian cancer cells and BGC-823 human gastric cancer cells as well as against HN-8, HNSCC, and HN-30 human head and neck squamous cell carcinoma cell lines [265,266]. It also decreased the cell viability and induced apoptosis in Saos-2 human osteosarcoma, T24 human bladder cancer, MCF-7 human breast cancer cells, and WM-266-4 human melanoma cells [267,268,269,270]. OXYRES inhibited growth and induced caspase-independent apoptosis-like cell death in chemoresistant, triple-negative MDA-MB-231 human breast cancer cells [271]. Moreover, OXYRES inhibited migration of HCT116 and HT-29 human colon cancer cells, as well as prevented H22 murine hepatocellular carcinoma growth and lymph node metastasis [272,273].

#### Neuroblastoma

OXYRES was shown to induce autophagy along with apoptosis in SH-SYSY human neuroblastoma cells (Table 2) [274]. The IC_50_ value of OXYRES estimated after 12 h of treatment of SH-SYSY cells with the compound was 140 µM. Treatment with OXYRES dose-dependently and time-dependently increased autophagy as determined by the immunoblot detection of increased protein levels of Beclin 1, LC3-II, ATG5, and ATG7. Activation of autophagy in SH-SYSY cells was also confirmed by fluorescence microscopy after staining with dansylcadaverine, a marker specific for AVOs, as well as after staining with anti-LC3 antibodies. AVOs were also detected after AO staining followed by the flow cytometric analysis. Moreover, OXYRES induced apoptosis in SH-SYSY cells, as revealed by the detection of morphological changes in cells, as well as phosphatidylserine externalization, mitochondrial membrane depolarization, upregulation of Bax/Bcl-2 ratio, and activation of caspase-3 and caspase-9. Furthermore, both autophagy and apoptosis appeared to contribute to OXYRES cytotoxicity against SH-SYSY cells but were independent of each other. The autophagy inhibitor, 3-MA, significantly reduced autophagy in SH-SYSY cells but did not inhibit apoptosis. In addition, in the presence of 3-MA OXYRES-induced cytotoxicity was significantly but not completely reduced. Similarly, in the presence of caspase-3 inhibitor Z-DEVD-FMK, the cytotoxicity of the compound significantly but not completely decreased. Neither inhibition of caspase-3 with Z-DEVD-FMK nor downmodulation of the *CASP3* gene with siRNA could affect autophagy in OXYRES-treated SH-SYSY cells. Moreover, it was found that the effects of OXYRES in SH-SYSY cells were related to the inhibition of PI3K/AKT/mTOR/pS6 signaling and activation of the p38 MAPK pathway [274].

### 6.5. PIN

PIN has been reported to exhibit anti-oxidant, anti-microbial, anti-fungal, and anti-inflammatory properties [64,275,276]. It has also been found to inhibit the cell growth of human oral, colon, and prostate cancer cell lines [277,278,279,280].

#### Leukemia

PIN at the concentration of 100 µM was shown to trigger apoptosis and autophagy in THP-1 human monocytic leukemia and U937 human pro-monocytic myeloid leukemia cells (Table 2) [281]. Treatment of THP-1 and U937 cells with PIN resulted in caspase-3 activation and phosphatidylserine externalization, indicative of apoptotic cell death. PIN also led to the conversion of LC3 protein from LC3-I to LC3-II in both studied cell lines. These effects were consistent with the observed increased number of LC3-positive fluorescent puncta in PIN-treated GFP-LC3-transfected THP-1 and U937 cells. Interestingly, treatment of THP-1 and U937 cells with 100 µM PIN for 4 and 6 h decreased p62/SQSTM1 protein level, suggesting degradation of this protein during autophagy. However, longer incubation with 100 µM PIN, lasting 12 or 24 h, resulted in an increase in p62/SQSTM1 protein level with a concomitant increase in LC3-II protein level. Additionally, autophagy inhibitors leupeptin and MHY1485 as well as caspase-3 inhibitor Ac-DEVD-CHO decreased the cytotoxic effects of PIN in both studied cell lines as revealed by the cytotoxicity assay. Furthermore, the AMPKα protein was found to play an important role in PIN-induced autophagy and apoptosis in THP-1 and U937 cells. PIN promoted AMPKα phosphorylation. However, PIN treatment decreased AMPKα protein levels in both cell lines.

### 6.6. CA-4

Combretastatins are a group of compounds comprising stilbenes (combretastatins A), dihydrostilbenes (combretastatins B), phenanthrenes (combretastatins C), and macrocyclic lactones (combretastatins D) [282]. CA-4 is a bioactive cis-stilbene that exhibits anti-cancer activity, acting as an inhibitor of tubulin polymerization and a vascular targeting agent [74,283]. To circumvent the low water solubility of CA-4, its prodrug combretastatin A-4 phosphate has been designed. Combretastatin A-4 phosphate has been tested in both preclinical and clinical trials [282].

#### Osteosarcoma

CA-4 was shown to induce autophagy in SJSA and MG63.2 human osteosarcoma cells (Table 2) [284]. Treatment of SJSA and MG63.2 cells with various concentrations of CA-4 caused a dose-dependent activation of autophagy as evidenced by the conversion of LC3-I to LC3-II and a decrease in the p62/SQSTM1 protein level. The LC3-II level increased in the presence of the autophagy inhibitor CQ, indicating an increase in autophagic flux following treatment with CA-4. Moreover, after exposure to CA-4, in SJSA and MG63.2 cells stably expressing GFP-tagged LC3 an increased number of GFP-LC3-positive fluorescent puncta was observed, corresponding to the accumulation of autophagosomes. Importantly, CA-4 and CQ combination treatment exerted a synergistic effect in inducing apoptosis in SJSA and MG63.2 cells as shown by the detection of the sub-G0/G1 cell population, PARP1 cleavage and caspase-3, -8, -9 activation. This finding also indicates that autophagy induced by CA-4 in SJSA and MG63.2 cells played a pro-survival role.

**Table 2:** Autophagy accompanied by apoptosis in human cancer cell lines treated by stilbenes.

Compound	Cell Line	Effects		Compound Concentration/ Treatment Time	Ref.
RES	A549	*Autophagy:* ↑ LC3-II/LC3-I (WB) ↑ Beclin-1 (WB) ↓ p62/SQSTM1 (WB) LC3-GFP fluorescent puncta (FM)	*Apoptosis:* phosphatidylserine externalization (FC)	25, 100, 200 µM	[165]
		↓ p-Akt/Akt (WB), ↓ p-mTOR/mTOR (WB) ↑ p-p38/p38 (WB), ↓ p-p70S6K/p70S6K (WB)			
	A549	*Autophagy:* ↑ LC3-II/LC3-I (WB) ↑ Beclin 1 (WB) ↓ p62/SQSTM1 (WB) MDC fluorescent puncta (FM) accumulation of autophagic vacuoles (TEM)	*Apoptosis:* phosphatidylserine externalization (FC) DNA fragmentation (FC) caspase-3 cleavage (WB) ↑ Bax (WB) ↓ Bcl-2 (WB)	25, 50, 100 µM/48 h	[166]
		↓ p-Akt/Akt (WB), ↑ p53 (WB), ↓ p-MDM2 (WB)			
	A549	*Autophagy:* ↑ LC3-II (WB) LC3 fluorescent puncta (CM)	*Apoptosis:* phosphatidylserine externalization (FC) ↓ mitochondrial membrane potential (CM) PARP-1 cleavage (WB) caspase-3 cleavage (WB) caspase-3 activity (C) ↑ Bax (WB), ↓ Bcl-2 (WB) cytochrome c release (WB)	100, 200, 300 µM/6, 12, 24 h	[167]
	A549	*Autophagy:* ↑ LC3-II/LC3-I (WB) ↑ Beclin 1 (WB) RFP-GFP-LC3 fluorescent puncta (CM) MDC fluorescent puncta (FM) ↑ AVOs (FC) accumulation of autophagic vacuoles (TEM)	*Apoptosis:* phosphatidylserine externalization (FC) DNA fragmentation (FC) ↑ Bax (WB) ↓ Bcl-2 (WB)	25, 30, 50, 75, 80 µM/48 h	[168]
		↑ NGFR (WB), ↑ p-AMPK/AMPK (WB), ↓ p-mTOR/mTOR (WB)			
	PC9/G /gefitinib resistant	*Autophagy:* ↑ LC3-II (WB) MDC fluorescent puncta (FM, FC)	*Apoptosis:* phosphatidylserine externalization (FC) condensation and fragmentation of nuclei (FM) caspase-3 cleavage (WB)	40 µM/72 h	[169]
		↑ p53 (WB), ↑ p21 (WB)			
	CAR/ cisplatin resistant	*Autophagy:* ↑ LC3-II (WB) ↑ Beclin 1 (WB) ↑ ATG5 (WB), ↑ ATG7 (WB) ↑ ATG12 (WB) ↑ ATG14 (WB) ↑ ATG16L1 (WB) AVOs (FM) LC3-GFP fluorescent puncta (FM) MDC fluorescent puncta (FM) LysoTracker Red DND-99 fluorescent puncta (FM)	*Apoptosis:* DNA condensation and fragmentation (FM) caspase-3 cleavage (WB) caspase-9 cleavage (WB) ↑ cytochrome c (WB) ↑ Apaf-1 (WB), ↑ AIF (WB) ↑ Endo G (WB) ↑ Bax (WB), ↑ Bad (WB) ↓ Bcl-2 (WB), ↓ p-Bad (WB)	25, 50, 100 µM/24, 48 h	[170]
		↑ p-AMPK (WB), ↓ p-Akt (WB), ↓ p-mTOR (WB) ↑ PI3K class III (WB), ↓ Rubicon (WB)			
	EC109	*Autophagy:* ↑ LC3-II (WB), ↑ Beclin 1 (WB) ↑ ATG5 (WB) LC3 fluorescent puncta (FM) MDC fluorescent puncta (FM) accumulation of autophagic vacuoles (TEM)	*Apoptosis:* phosphatidylserine externalization (FC) ↑ sub-G0/G1 (FC) ↓ mitochondrial membrane potential (FC) caspase-3 cleavage (WB) caspase-3 activation (C) ↑ Bax (WB), ↓ Bcl-2 (WB) chromatin condensation (FM)	10, 50, 100, 150 µM/6, 12, 24, 48 h	[171]
		↑ p-AMPK (WB)			
	EC9706	*Autophagy:* ↑ LC3-II (WB), ↑ Beclin-1 (WB) ↑ ATG5 (WB) LC3 fluorescent puncta (FM) MDC fluorescent puncta (FM)	*Apoptosis:* phosphatidylserine externalization (FC) ↑ sub-G0/G1 (FC) caspase-3 cleavage (WB) ↑ Bax (WB), ↓ Bcl-2 (WB) chromatin condensation (FM)	10, 50, 100, 150 µM/6, 12, 24, 48 h	[171]
		↑ p-AMPK (WB)			
	HT-29	*Autophagy:* ↑ LC3-II (WB) accumulation of autophagic vacuoles (TEM) LC3 fluorescent puncta (CM)	*Apoptosis:* phosphatidylserine externalization (FC) caspase-3 cleavage (WB) caspase-8 cleavage (WB) PARP cleavage (WB)	150 µM/24, 48, 72 h	[172]
	DLD1	*Autophagy:* ↑ LC3-II (WB), ↑ Beclin 1(WB) GFP–LC3 fluorescent puncta (FM) LC3-FITC fluorescent puncta (FM) Beclin 1–GFP fluorescent puncta (FM) Lamp-1 fluorescent puncta (FM) MDC fluorescent puncta (FM)	*Apoptosis:* phosphatidylserine externalization (FC) DNA fragmentation (FM)	100 µM/15 min, 1, 2, 4, 24, 48 h	[173]
	MDA-MB231	*Autophagy:* ↑ LC3-II (WB), ↑ Beclin 1 (WB)	*Apoptosis:* caspase-3 cleavage (WB) caspase-3 activation (SF)	50, 60, 120 µM/12, 24, 36, 48 h	[174]
	A2780	*Autophagy:* accumulation of autophagic vacuoles (TEM) MDC fluorescent puncta (FM)	*Apoptosis:* caspase-9 cleavage (WB) cytochrome c release (WB)	25, 50, 100 µM/6, 12, 24, 36, 48 h	[175]
	OVCAR-3	*Autophagy:* ↑ ATG5 (WB), ↑ LC3-II (WB) ↑ p62/SQSTM1 (WB)	*Apoptosis:* phosphatidylserine externalization (FC) ↓ mitochondrial membrane potential (FC, FM) caspase-3 activation (C, CM) caspase-3 cleavage (WB) ↑ PARP activity (C)	30, 100 µM /12, 24, 48, 72 h	[176]
	HeLa	*Autophagy:* ↑ LC3-II (WB) LC3-GFP fluorescent puncta (FM) MDC fluorescent puncta (FM) accumulation of autophagic vacuoles (TEM)	*Apoptosis:* phosphatidylserine externalization (FC) caspase-3 cleavage (WB) cytochrome c release (WB)	100 µM/6, 12, 24, 48, 72 h	[177]
	Ishikawa	*Autophagy:* ↑ LC3-II (WB) LC3 fluorescent puncta (CM)	*Apoptosis:* phosphatidylserine externalization (FC) ↑ sub-G1 fraction (FC) PARP cleavage (WB)	25, 100 µM/24, 48, 72 h	[178]
		↑ p-AMPKα (WB), ↑ p-ERK (WB), ↓ p-AKT (WB)			
	786-O	*Autophagy:* ↑ LC3-II (WB) accumulation of autophagic vacuoles (FC, FM)	*Apoptosis:* phosphatidylserine externalization (FC) ↓ mitochondrial membrane potential (FC) caspase-3 activation (FC) PARP cleavage (WB)	20, 40 µM/24, 48 h	[179]
		↓ p-ERK/ERK (WB), ↑ p-JNK/JNK (WB), ↑ p-p38/p38 (WB)			
	U251	*Autophagy:* ↑ LC3-II (WB) ↑ Beclin 1 (WB) LC3 fluorescent puncta (CM) MDC fluorescent puncta (FM)	*Apoptosis:* ↑ sub-G1 (FC) ↓ mitochondrial membrane potential (FC, FM), chromatin condensation and nuclei fragmentation (FM)	150 µM/3, 6, 12, 24, 48, 72 h	[180]
	K562	*Autophagy:* ↑ LC3-II (WB) accumulation of autophagic vacuoles (TEM)	*Apoptosis:* phosphatidylserine externalization (FC) caspase-3 activation (C) fragmentation of nuclei (FM)	12.5, 25, 50, 100 µM/4 days	[181]
		↑ glycophorin A (FC), ↑ CD71 (FC), ↑ Band3 (FC)			
	K562 /imatinib-sensitive	*Autophagy:* ↑ LC3-II (WB) ↑ p62/SQSTM1 (WB) accumulation of autophagic vacuoles (TEM)	*Apoptosis:* caspase-3 activation (SF)	2, 5, 10, 25, 50 µM/2, 4, 8, 16, 24, 48 h	[182]
		↑ p-AMPKα(T172) (WB), ↓ p-mTOR(S2448) (WB) ↓ p-p70/85-S6K (T389) (WB), ↓ p-S6 ribosomal (S235/236) (WB) ↓ p-4EBP1 (T37/46) (WB), ↑ p-JNK 2/3 (T183/Y185) (WB) ↑ p-JNK 1 (T183/Y185) (WB), ↑ p-c-Jun (S63) (WB)			
	K562 /imatinib-resistant	*Autophagy:* ↑ LC3-II (WB), ↑ p62/SQSTM1 (WB) accumulation of autophagic vacuoles (TEM)	*Apoptosis:* caspase-3 activation (SF)	2, 5, 10, 25, 50 µM/2, 4, 8, 16, 24, 48 h	[182]
		↑ p-AMPKα(T172) (WB), ↓ p-mTOR(S2448) (WB) ↓ p-p70/85-S6K (T389) (WB), ↓ p-S6 ribosomal (S235/236) (WB) ↓ p-4EBP1 (T37/46) (WB), ↑ p-JNK 2/3 (T183/Y185) (WB) ↑ p-JNK 1 (T183/Y185) (WB), ↑ p-c-Jun (S63) (WB)			
	K562/ adriamycin resistant	*Autophagy:* ↑ LC3-II (WB), ↑ Beclin 1 (WB) ↓ p62/SQSTM1 (WB) ↑ cathepsin D (WB) accumulation of autophagic vacuoles (TEM) MDC fluorescent puncta (FM)	*Apoptosis:* phosphatidylserine externalization (FC) caspase-3 cleavage (WB) ↓ Bcl-2 (WB), ↑ Bax (WB)	40, 80 µM/24, 48, 72 h	[183]
	MOLT-4	*Autophagy:* ↑ LC3-II (WB) ↑ p62/SQSTM1 (WB) LC3 fluorescent puncta (FM)	*Apoptosis:* caspase-3 activation (FC) ↓ mitochondrial membrane potential (FC) phosphatidylserine externalization (FC) ↑ sub-G1 (FC) PARP1 cleavage (WB) DNA fragmentation (AGE) condensation and fragmentation of nuclei (FM)	41 µM/45 min, 2, 4, 6, 12, 24, 48 h	[184]
	HL-60	*Autophagy:* ↑ LC3-II (WB) ↑ p62/SQSTM1 (24 h) (WB) ↓ p62/SQSTM1 (48 h, 72 h) (WB) LC3 fluorescent puncta (FM)	*Apoptosis:* ↑ caspase-3 activation (FC) ↓ mitochondrial membrane potential (FC) phosphatidylserine externalization (FC) ↑ sub-G1 (FC) PARP1 cleavage (WB) DNA fragmentation (AGE) condensation and fragmentation of nuclei (FM)	43 µM/45 min, 2, 4, 6, 12, 24, 48, 72 h	[184]
	HL-60	*Autophagy:* ↑ LC3-II (WB) ↑ p62/SQSTM1(WB) ↑ Beclin-1 (WB) ↑ ATG5 (WB) LC3 fluorescent puncta (FM)	*Apoptosis:* ↓ mitochondrial membrane potential (SF) phosphatidylserine externalization (FC) caspase-3 cleavage (WB) ↑ caspase-3 activation (C) caspase-8 cleavage (WB) Bid cleavage (WB) ↑ Bax/Bcl-2 (WB), ↑ Fas (WB) ↑ FasL (WB)	12,5, 25, 50, 100 µM/3, 6, 12, 24, 48 h	[185]
		↑ p-AMPK/AMPK (WB), ↑ pLKB1/LKB1 (WB) ↓ p-AKT/AKT(WB), ↓ p-p70S6K/p70S6K (WB)			
	U266	*Autophagy:* ↑ LC3-II (WB), ↑ Beclin 1 (WB)	*Apoptosis:* phosphatidylserine externalization (FC) caspase-3 cleavage (WB) PARP cleavage (WB) ↓ Survivin (WB)	50, 100 µM/48 h	[186]
		↑ p-AMPKα (WB), ↓ p-mTOR (WB) ↓ p-p70S6K (WB), ↓ p-4EBP1 (WB)			
	RPMI-8226	*Autophagy:* ↑ LC3-II (WB), ↑ Beclin 1 (WB)	*Apoptosis:* phosphatidylserine externalization (FC) caspase-3 cleavage (WB) PARP cleavage (WB) ↓ Survivin (WB)	50, 100 µM/48 h	[186]
		↑ p-AMPKα (WB), ↓ p-mTOR (WB) ↓ p-p70S6K (WB), ↓ p-4EBP1 (WB)			
	NCI-H929	*Autophagy:* ↑ LC3-II (WB), ↑ Beclin 1 (WB)	*Apoptosis:* phosphatidylserine externalization (FC) caspase-3 cleavage (WB) PARP cleavage (WB) ↓ Survivin (WB)	50, 100 µM/48 h	[186]
		↑ p-AMPKα (WB), ↓ p-mTOR (WB) ↓ p-p70S6K (WB), ↓ p-4EBP1 (WB)			
PTER	A549	*Autophagy:* ↑ LC3-II (WB), ↑ Beclin 1 (WB) ↓ p62/SQSTM1 (WB) GFP-LC3 fluorescent puncta (FM) ↑ AVOs (FC)	*Apoptosis:* phosphatidylserine externalization (FC) ↑ sub-G0/G1 (FC) condensed and fragmented nuclei (FM)	50, 75, 100 µM/ 24, 48, 72 h	[221]
		↓ p-PI3K (WB), ↓ p-AKT (WB) ↓ p-JNK (WB), ↑ p-ERK (WB)			
	A549/D16 /docetaxel resistant	*Autophagy:* ↑ LC3-II (WB), ↑ Beclin 1 (WB) ↓ p62/SQSTM1 (WB) GFP-LC3 fluorescent puncta (FM) ↑ AVOs (FC)	*Apoptosis:* phosphatidylserine externalization (FC) ↑ sub-G0/G1 (FC) condensed and fragmented nuclei (FM)	50, 75, 100 µM/24, 48, 72 h	[221]
		↓ p-PI3K (WB), ↓ p-AKT (WB) ↓ p-JNK (WB), ↑ p-ERK (WB)			
	SAS	*Autophagy:* ↑ LC3-II (WB), ↑ Beclin 1 (WB) GFP-LC3 fluorescent puncta (FM) ↑ AVOs (FC, FM)	*Apoptosis:* phosphatidylserine externalization (FC) caspase-3 cleavage (WB) caspase-8 cleavage (WB) caspase-9 cleavage (WB) PARP cleavage (WB) condensed and fragmented nuclei (FM)	10, 20, 40 µM/6, 12, 18, 24, 48 h	[222]
		↓ pp-38 (WB), ↓ p-AKT (WB), ↓ p-ERK1/2(WB) ↓ p-mTOR (WB), ↓ p-ULK (Ser757) (WB), ↑ p-ULK (Ser555) (WB) ↑ p-JNK1/2 (WB), ↑ p-AMPK (WB), ↑ p-Raptor (WB)			
	OECM-1	*Autophagy:* ↑ LC3-II (WB), ↑ Beclin 1 (WB) GFP-LC3 fluorescent puncta (FM) ↑ AVOs (FC, FM)	*Apoptosis:* phosphatidylserine externalization (FC) caspase-3 cleavage (WB) caspase-8 cleavage (WB) caspase-9 cleavage (WB) PARP cleavage (WB) condensed and fragmented nuclei (FM)	10, 20, 40 µM/6, 12, 18, 24, 48 h	[222]
		↓ pp-38 (WB), ↓ p-AKT (WB), ↓ p-ERK1/2 (WB), ↓ p-mTOR (WB) ↓ p-ULK (Ser757) (WB), ↑ p-ULK (Ser555) (WB) ↑ p-JNK1/2 (WB), ↑ p-AMPK (WB), ↑ p-Raptor (WB)			
	CAR/ cisplatin resistant	*Autophagy:* ↑ LC3-II (WB), ↑ Beclin 1 (WB) ↑ ATG5 (WB), ↑ ATG7 (WB) ↑ ATG12 (WB) ↑ AVOs (FM) MDC fluorescent puncta (FM)	*Apoptosis:* caspase-3 activation (C) caspase-9 activation (C) caspase-3 cleavage (WB) caspase-7 cleavage (WB) caspase-9 cleavage (WB) PARP cleavage (WB) ↓ Bcl-2 (WB), ↑ Bax (WB) ↑ cytochrome c (WB) DNA fragmentation (FC) chromatin condensation (FM)	25, 50, 75, 100 µM/24, 48 h	[223]
		↓ p-AKT (WB), ↓ MDR1 (WB)			
	HT-29	*Autophagy:* ↑ *ULK1* (qRT-PCR) ↑ *AMBRA1* (qRT-PCR) ↑ *MAP1LC3A* (qRT-PCR)	*Apoptosis:* caspase-3 activation (C) ↑ sub-G1 (FC) DNA fragmentation (C) ↑ *BAX* (qRT-PCR)	10, 40, 60 µM/6, 12, 24, 48, 72 h	[224]
		↓ p-AKT/total AKT (C), ↓ p-STAT/total STAT (C)			
	BxPC-3	*Autophagy:* ↑ LC3-II (WB), ↑ Beclin 1 (WB) ↑ p62/SQSTM1 (WB) ↑ AVOs (FM, FC)	*Apoptosis:* phosphatidylserine externalization (FC) ↑ sub-G0/G1 (FC) caspase-3 cleavage (WB) ↓ Bcl-XL (WB), ↓ Bcl-2 (WB)	75, 100, 125µM/12, 24, 36, 48 h	[225]
		↓ p-AKT (WB), ↓ p-mTOR (WB), ↑ p-ERK (WB) ↓ p-p38 (WB), ↓ p-JNK (WB)			
	MIA PaCa-2	*Autophagy:* ↑ LC3-II (WB), ↑ Beclin 1 (WB) ↑ p62/SQSTM1 (WB) ↑ AVOs (FM, FC) accumulation of autophagic vacuoles (TEM)	*Apoptosis:* phosphatidylserine externalization (FC) ↑ sub-G0/G1 (FC) caspase-3 cleavage (WB) caspase-8 cleavage (WB) caspase-9 cleavage (WB) ↓ Bcl-XL (WB), ↓ Bcl-2 (WB) ↑ Bax (WB)	75, 100, 125 µM/12, 24, 36, 48 h	[225]
		↓ p-AKT (WB), ↓ p-mTOR (WB), ↑ p-ERK (WB)			
	MCF-7	*Autophagy:* ↑ LC3-II (WB)	*Apoptosis:* phosphatidylserine externalization (FC) PARP cleavage (WB) chromatin condensation (FM)	50, 100 µM/24 h	[226]
	Bcap-37	*Autophagy:* ↑ LC3-II (WB)	*Apoptosis:* phosphatidylserine externalization (FC) PARP cleavage (WB) chromatin condensation (FM)	50, 100 µM/24 h	[226]
	T24	*Autophagy:* ↑ LC3-II (WB) ↑ AVOs (FC) accumulation of autophagic vacuoles (TEM)	*Apoptosis:* phosphatidylserine externalization (FC) ↑ sub-G0/G1 (FC) caspase-3 activation (SF) chromatin condensation and nuclei fragmentation (FM) ↓ Bcl-2 (WB), ↓ Bcl-XL (WB)	50, 75, 100 µM/24, 48, 72 h	[227]
		↓ p-AKT (WB), ↓ pp70S6K (WB), ↑ p-ERK1/2 (WB)			
	T24R/ chemoresistant	*Autophagy:* ↑ LC3-II (WB) ↑ AVOs (FC) accumulation of autophagic vacuoles (TEM)	*Apoptosis:* phosphatidylserine externalization (FC) ↑ sub-G0/G1 (FC) caspase-3 activation (SF) chromatin condensation and nuclei fragmentation (FM) ↓ Bcl-2 (WB), ↓ Bcl-XL (WB)	50, 75, 100 µM/24, 48, 72 h	[227]
	HL-60	*Autophagy:* ↑ LC3-II (WB) LC3 fluorescent puncta (CM) accumulation of autophagic vacuoles (TEM)	*Apoptosis:* phosphatidylserine externalization (FC) ↑ sub-G1 (FC) DNA fragmentation (AGE) ↓ mitochondrial membrane potential (FC) caspase-3 activation (FC) chromatin condensation and nuclei fragmentation (CM)	1, 10, 43, 100 µM/2, 4, 6, 12, 24, 48, 72 h	[228]
PIC	SH-SY5Y	*Autophagy:* accumulation of autophagic vacuoles (FM) ↑ *BECN1* (qRT-PCR) ↑ *ATG5* (qRT-PCR) ↑ *ATG7* (qRT-PCR) ↑ *ATG12* (qRT-PCR) ↑ *MAP 1LC3A* (qRT-PCR) ↑ *MAP 1LC3B* (qRT-PCR)	*Apoptosis:* phosphatidylserine externalization (FC) DNA fragmentation (C) ↓ mitochondrial membrane potential (FM) ↑ *CASP3* (qRT-PCR) ↑ *CASP8* (qRT-PCR) ↑ *CASP9* (qRT-PCR) ↑ *BAX* (qRT-PCR) ↑ *FADD* (qRT-PCR)	50, 100 µM/72 h	[257]
	MOLT-4	*Autophagy:* ↑ LC3-II (WB) ↓ p62/SQSTM1 (WB) LC3 fluorescent puncta (FM)	*Apoptosis:* phosphatidylserine externalization (FC) ↑ sub-G1 (FC) DNA fragmentation (AGE) PARP1 cleavage (WB) chromatin condensation and nuclei fragmentation (FM) ↓ mitochondrial membrane potential (FC) caspase-3 activation (FC)	45.5 µM/ 45 min, 2, 4, 6, 12, 24, 48, 72, 96 h, 3 x 96 h	[258]
		↑ P-gp (FC), ↓ BCRP (FC)			
OXYRES	SH-SY5Y	*Autophagy:* ↑ LC3-II (WB), ↑ Beclin 1 (WB) ↑ ATG5 (WB), ↑ ATG7 (WB) ↑ AVOs (FC, FM) LC3 fluorescent puncta (FM)	*Apoptosis:* phosphatidylserine externalization (FC) ↓ mitochondrial membrane potential (FC) ↓ Bcl-2 (WB), ↑ Bax (WB) caspase-3 cleavage (WB) caspase-9 cleavage (WB) fragmented nuclei (FM)	40, 80, 120, 160 µM/2, 4, 6, 8, 10, 12 h	[274]
		↓ p-AKT (WB), ↓ p-mTOR (WB), ↓ p-S6 (WB) ↑ p-p38 (WB), ↓ p-ERK1/2 (WB)			
PIN	THP-1	*Autophagy:* ↑ LC3-II (WB) ↓ p62/SQSTM1, 4, 6 h (WB) ↑ p62/SQSTM1, 12, 24 h (WB) LC3 fluorescent puncta (FM)	*Apoptosis:* phosphatidylserine externalization (FC) caspase-3 cleavage (WB) caspase-3 activation (SF)	0.1, 1, 10, 50, 100 µM/4, 6, 12, 24 h	[281]
		↑ p-AMPKα (WB), ↓ AMPKα (WB)			
	U937	*Autophagy:* ↑ LC3-II (WB) ↓ p62/SQSTM1, 4, 6 h (WB) ↑ p62/SQSTM1, 12, 24 h (WB) LC3 fluorescent puncta (FM)	*Apoptosis:* phosphatidylserine externalization (FC) caspase-3 cleavage (WB) caspase-3 activation (SF)	0.1, 1, 10, 50, 100 µM/4, 6, 12, 24 h	[281]
		↓ AMPKα (WB)			
CA-4	SJSA	*Autophagy:* ↑ LC3-II (WB) ↓ p62/SQSTM1 (WB) GFP-LC3 fluorescent puncta (CM)	*Apoptosis:* ↑ sub-G0/G1 (FC) caspase-3 cleavage (WB) caspase-8 cleavage (WB) caspase-9 cleavage (WB) PARP cleavage (WB)	1, 5, 10 nM/24, 48, 72 h	[284]
	MG63.2	*Autophagy:* ↑ LC3-II (WB) ↓ p62/SQSTM1 (WB) GFP-LC3 fluorescent puncta (CM)	*Apoptosis:* ↑ sub-G0/G1 (FC) caspase-3 cleavage (WB) caspase-8 cleavage (WB) caspase-9 cleavage (WB) PARP cleavage (WB)	1, 5, 10 nM/24, 48, 72 h	[284]

WB—Western blotting; FC—flow cytometry; CM—confocal microscopy; FM—fluorescence microscopy; TEM—transmission electron microscopy, C—colorimetry; AGE—agarose gel electrophoresis; SF—spectrofluorymetry; qRT-PCR—quantitative real-time polymerase chain reaction.

## 7. Clinical Perspectives of Stilbenes in Cancer Therapy

Numerous in vivo studies have demonstrated the anti-cancer activity of stilbenes [155,187,188,189,190,229,230,231,232,259,260,261]. Some of the studies have shown that mechanisms of action of stilbenes in animal models may involve apoptosis [188,232,259] or both autophagy and apoptosis [187,189]. Clinical trials related to the application of stilbenes in anti-cancer therapy focused mainly on RES (https://clinicaltrials.gov NCT00920803, NCT01476592, NCT00256334, NCT00433576, NCT00920556, NCT03482401) and PTER (https://clinicaltrials.gov NCT03671811). The experimental data indicate that RES may be a promising chemotherapeutic agent in the treatment of colorectal cancer [285,286]. The results show that RES exhibits low RES bioavailability in humans due to its rapid metabolism [285]. However, accumulating evidence indicates that RES bioavailability may be improved by developing its new formulations such as micronized formulations or nano-formulations [285]. Further research is needed on the anti-cancer effects of new formulations of RES and other stilbenes and their combination with other drugs. To date, the low bioavailability of RES and other stilbenes limits their use in cancer treatment as neo-adjuvant or adjuvant chemotherapeutics. However, their use in the oncology field may be considered in patients after completion of anti-cancer therapy, especially when neo-adjuvant chemo/radiotherapy is followed by the surgical removal of the tumor. In agreement with the cancer stem cells theory, even single cells are able to reconstitute cancer cell populations [287]. Natural compounds may be used as a complementary therapy to help eliminate cancer cells remaining after surgery and thereby decrease the risk of cancer recurrence. Taking into account that RES exhibits anti-oxidant and immunomodulatory activities, it appears as a promising complementary drug. The supplementation with stilbenes in the oncology field may also be considered. However, clinical data are required to assess stilbenes efficacy and safety and gain acceptance among clinicians and patients.

## 8. Conclusions and Future Directions

Many stilbenes have been demonstrated to be promising chemopreventive and chemotherapeutic agents that exhibit pleiotropic mechanisms of action. Many of them have been reported to trigger both apoptotic and autophagic molecular pathways in different cancer types. Autophagy may act as a pro-survival or pro-death mechanism in cancer cells. Moreover, the chemoresistance of cancer cells may be facilitated or suppressed by autophagy depending on the context. Therefore, it is important to examine the interplay between autophagy and apoptosis and evaluate its role in drug-treated cancer cells. Modulation of autophagy may lead to the improvement of the efficacy of anti-cancer therapy. Inhibition of pro-survival autophagy or induction of autophagy-dependent cell death may increase the sensitivity of cancer cells to chemotherapeutics. In line with the above statements, the evaluation of drugs’ effects on autophagy deserves further attention. In the case of combination chemotherapy, commonly used in anti-cancer therapeutic strategies, it is important to find out whether drugs used in combination act synergistically in the context of autophagy and apoptosis.

## Figures and Tables

**Table 1:** Distribution of stilbenes in plants and plant-derived dietary products.

Stilbenes	Plants/Plant-Derived Dietary Products
RES	Grape [8,9,10,11,12], Grape cane [13,14], Grape juice [10,15,16] Red wine [9,16,17,18,19,20,21,22,23,24,25], White wine [9,18,19,20,21,23,25], Rose wine [9,19,21,23] Beer [26] Apple juice [10], Peach juice [10] Lowbush blueberry [8], Sparkleberry [8], Rabbiteye blueberry [8] Highbush blueberry [8], Elliott’s blueberry [8], Cranberry [8,16,27] Bilberry [8,27], Deerberry [8], Lingonberry [8] Partridgeberry [8], Cowberry [27], Mulberry [28] Strawberry [27], Red currant [27] Peanut [29,30], Peanut butter [29], Pistachio [30] Cocoa powder [31], Chocolate [31] Tomato [32] Green tea [10], Black tea [10], Red tea [10], Camomile [10] Rhubarb *Rheum undulatum* [33], Rhubarb *Rheum palmatum* [34] Eucalyptus [35,36] Spruce [37], Spruce *Picea jezoensis* [38], Spruce *Picea abies* [39] *Polygonum cuspidatum* [40,41] *Gnetum gnemon* [42], *Gnetum klossii* [43] *Stuhlmannia moavi* [44]
PTER	Grape [45,46], Grape cane [14] Rabbiteye blueberry [8], Deerberry [8] Peanut [47] *Pterocarpus marsupium* [48], *Pterocarpus santalinus* [49] *Pterocarpus tinctorius* [49] *Guibourtia tessmanii* [50]
PIC	Grape [9,10,11,12], Grape cane [13,14], Grape juice [10] Red wine [9,23,25], White wine [23,25], Rose wine [23] Apple juice [10], Peach juice [10] Highbush blueberry [8], Deerberry [8], Mulberry [51] Peanut [41] Passion fruit seeds [52] Green tea [10], Black tea [10], Red tea [10], Camomile [10] Rhubarb *Rheum undulatum* [33], Rhubarb *Rheum palmatum* [34] Rhubarb *Rheum rhaponticum* [53], Rhubarb *Rheum rhabarbarum* [53] Sugar cane [54] Spruce [37], Spruce *Picea jezoensis* [38], Spruce *Picea abies* [39] Eucalyptus [36] *Polygonum cuspidatum* [41] *Stuhlmannia moavi* [44]
OXYRES	Grape [55] Red wine [23], White wine [23], Rose wine [23] Mulberry [28,56] *Artocarpus lakoocha* [57] *Pterocarpus marsupium* [58]
GNE	*Gnetum gnemon* [42,59], *Gnetum montanum* [60], *Gnetum klossii* [43] *Gnetum macrostachyum* [61], *Gnetum microcarpum* [62] *Gnetum hainanense* [63]
PIN	*Pinus sylvestris* [64,65], *Pinus densiflora* [66], *Pinus resinosa* [67,68] *Pinus banksiana* [67,68], *Pinus strobus* [68], *Picea glauca* [67] *Gnetum cleistostachyum* [69]
RHAP	Rhubarb *Rheum emodi* [70], Rhubarb *Rheum palmatum* [34] Rhubarb *Rheum undulatum* [33], Rhubarb *Rheum officinale* [71] Rhubarb *Rheum rhaponticum* [53], Rhubarb *Rheum rhabarbarum* [53] *Caesalpinia sinensis* [72] *Stuhlmannia moavi* [44] *Gnetum hainanense* [63], *Gnetum cleistostachyum* [69]
ISORHAP	Grape [11], Grape cane [13] Spruce [37], Spruce *Picea jezoensis* [38], Spruce *Picea abies* [39] *Gnetum macrostachyum* [61], *Gnetum cleistostachyum* [69] *Gnetum gnemon* [42], *Gnetum klossii* [43], *Gnetum hainanense* [63] Rhubarb *Rheum rhaponticum* [53], Rhubarb *Rheum rhabarbarum* [53] *Stuhlmannia moavi* [44] *Saccharum spontaneum (wild sugar cane)* [73]
DEOXYRHAP	Rhubarb *Rheum emodi* [70], Rhubarb *Rheum palmatum* [34] Rhubarb *Rheum undulatum* [33], Rhubarb *Rheum rhaponticum* [53] Rhubarb *Rheum rhabarbarum* [53] *Gnetum cleistostachyum* [69]
CA-4	*Combretum caffrum* [74], *Combretum leprosum* [75]
